# Phenotypic evaluation and genetic dissection of resistance to *Phytophthora sojae* in the Chinese soybean mini core collection

**DOI:** 10.1186/s12863-016-0383-4

**Published:** 2016-06-18

**Authors:** Jing Huang, Na Guo, Yinghui Li, Jutao Sun, Guanjun Hu, Haipeng Zhang, Yanfei Li, Xing Zhang, Jinming Zhao, Han Xing, Lijuan Qiu

**Affiliations:** National Center for Soybean Improvement/National Key laboratory of Crop Genetics and Germplasm enhancement, Key laboratory of Biology and Genetics and Breeding for Soybean, Ministry of Agriculture, Nanjing Agricultural University, Nanjing, 210095 People’s Republic of China; The National Key Facility for Crop Gene Resources and Genetic Improvement (NFCRI)/Key Lab of Germplasm Utilization (MOA), Institute of Crop Science, Chinese Academy of Agricultural Sciences, 100081 Beijing, People’s Republic of China

**Keywords:** Soybean mini core collection, *Phytophthora sojae*, Resistance evaluation, Association mapping, Genetic diversity

## Abstract

**Background:**

Phytophthora root and stem rot (PRR) caused by *Phytophthora sojae* is one of the most serious diseases affecting soybean (*Glycine max* (L.) Merr.) production all over the world. The most economical and environmentally-friendly way to control the disease is the exploration and utilization of resistant varieties.

**Results:**

We screened a soybean mini core collection composed of 224 germplasm accessions for resistance against eleven *P. sojae* isolates. Soybean accessions from the Southern and Huanghuai regions, especially the Hubei, Jiangsu, Sichuan and Fujian provinces, had the most varied and broadest spectrum of resistance. Based on gene postulation, *Rps*1b, *Rps*1c, *Rps*4, *Rps*7 and novel resistance genes were identified in resistant accessions. Consequently, association mapping of resistance to each isolate was performed with 1,645 single nucleotide polymorphism (SNP) markers. A total of 14 marker-trait associations for *Phytophthora* resistance were identified. Among them, four were located in known PRR resistance loci intervals, five were located in other disease resistance quantitative trait locus (QTL) regions, and five associations unmasked novel loci for PRR resistance. In addition, we also identified candidate genes related to resistance.

**Conclusion:**

This is the first *P. sojae* resistance evaluation conducted using the Chinese soybean mini core collection, which is a representative sample of Chinese soybean cultivars. The resistance reaction analyses provided an excellent database of resistant resources and genetic variations for future breeding programs. The SNP markers associated with resistance will facilitate marker-assisted selection (MAS) in breeding programs for resistance to PRR, and the candidate genes may be useful for exploring the mechanism underlying *P. sojae* resistance.

**Electronic supplementary material:**

The online version of this article (doi:10.1186/s12863-016-0383-4) contains supplementary material, which is available to authorized users.

## Background

Phytophthora root and stem rot (PRR) caused by the oomycete pathogen *Phytophthora sojae* Kaufmann & Gerdemann brings about extensive economic losses worldwide [1]. The pathogen infects soybean throughout the growing season, resulting in seedling damping-off and root and stem rotting of mature plants. *P sojae* isolates are classified into pathotypes based on the reactions of soybean differentials carrying resistance genes, and more than two hundred pathotypes of *P. sojae* have been identified worldwide to date [2, 3]. In China, an aboriginal *P. sojae* isolate was first found in 1989 in Heilongjiang province [4, 5]. Since then, the emergence of new races and their virulence diversity have been reported continuously in other major soybean production areas around the Huanghuai valley and in southern China [6], implying that PRR is a potential threat to Chinese soybean production.

Deployment of *Rps* (resistance to *P. sojae*) genes in soybean cultivars has been an effective method of controlling PRR [7] because *Rps* genes mediate complete or race-specific resistance. To date, at least twenty *Rps* genes that protect against *P. sojae* infection have been identified in soybean [8–14]. The efficacies of these *Rps* genes are dependent on the presence of corresponding avirulence (*Avr*) genes in the pathogen. The *Rps* genes recognize *Avr* genes secreted from *P. sojae* in a gene-for-gene model, thus activating the plant resistant response [15, 16]. Under the theory of pathogen-host coevolution [17], *P. sojae* is under strong selection pressure, and new pathotypes are constantly evolving. Therefore, the *Rps* genes is easily overcome by changes in the virulence of *P. sojae* [16].

Normally, a single *Rps* gene is effective for only 8 to 15 years [18]. The first *Rps* gene *Rps*1k was identified in 1957 [8]; to date, no single *Rps* gene has been found to confer resistance to all *P. sojae* isolates, and new virulent pathotypes of *P. sojae* have continued to emerge. Mining of novel resistance genes has thus become an urgent priority in soybean resistance breeding. Many surveys on resistant germplasm characterization and resistance gene discovery in the Chinese soybean have been performed [19–22]. In these surveys, soybean germplasm accessions were randomly selected and concentrated in certain regions, such as central China [19], southern China [20] and the Huanghuai region [21, 22].

Chinese soybean germplasm resources are very vast, making it difficult to thoroughly evaluate *Phytophthora* resistance; so far, no systematic screening of countrywide soybean germplasm for resistance has been carried out. The soybean mini core collection is a preferred choice for efficient exploration of variations in genetic resources because it represents 1 % of the entire GeneBank collection but 94.5 % of the soybean phenotypic diversity and 63.5 % of the genetic diversity of the collection [23]. The soybean mini core collection has been used to evaluate desirable traits, such as protein subunit variations and seed quality traits, and some of the mini core collection accessions have been used to identify disease phenotype, such as soybean cyst nematode (SCN) and soybean mosaic virus (SMV) [24]. Recently, variants partial resistant to *P. sojae* in the mini core collection were also characterized [25].

Association mapping is a method used to identify markers associated with a particular trait by using linkage disequilibrium (LD) between alleles within natural populations [26], with no need for developing biparental populations [27]. Recently, association mapping has been used to identify marker-trait associations in higher plants [28, 29], including various disease resistance associations in soybean, such as sclerotinia stem rot (SSR) resistance [30, 31] and sudden death syndrome (SDS) resistance [32]. The mini core collection, most of which consists of local landraces with alleles left behind during the processes of domestication and positive selection [24], may be an ideal population for association mapping analysis.

Here, we report phenotypic and genotypic screening of the Chinese soybean mini core collection for resistance to eleven *P. sojae* isolates with varying virulence. Our aim is to investigate the distribution of *Phytophthora* resistant germplasm and provide a new resistance gene pool for future breeding programs. An association analysis was developed to identify markers associated with *Phytophthora* resistance in the mini core collection, and the results will be useful for genomic identification of loci conferring resistance to *P. sojae* and for exploration of the genetic basis of resistance.

## Methods

### Plant materials

The soybean mini core collection is a set of 248 accessions chosen from 23,587 germplasm accessions conserved in the Chinese National Soybean GeneBank (CNSGB), which effectively maintains the genetic diversity of soybean in China. The accessions were provided by Professor Lijuan Qiu of the Chinese Academy of Agriculture Sciences. Excluding those without SNP marker data, 224 accessions from the mini core collection were used in this study. The collection consists of 196 landraces and 28 modern cultivars originating from 26 provinces and spread across four ecological regions representing four major soybean planting areas as follows: the North region (NR), the Northeast region (NER), the Huanghuai region (HHR) and the South region (SR) (Additional file 1).

A set of 13 differential cultivars, each possessing a single *Rps* gene, and one susceptible cultivar without any known *Rps* genes were used to confirm the pathotypes of the *P. sojae* isolates. These cultivars were Harlon (*Rps*1a), Harosoy13XX *(Rps*1b), Williams79 (*Rps*1c), PI103091 (*Rps*1d), Williams82 (*Rps*1k), L76-1988 (*Rps*2), Chapman (*Rps*3a), PRX146-36 (*Rps*3b), PRX145-48 (*Rps*3c), L85-2352 (*Rps*4), L85-3059 (*Rps*5), Harosoy62XX (*Rps*6), Harosoy (*Rps*7) and Williams (susceptible). The seeds were stored in our laboratory.

### Pathogen isolate virulence

The 11 *P. sojae* isolates (P6497, HLJ08-17, P7063, AH, H15, HeN08-35, PNJ1, Pmg, Pm28, Pm31 and JS08-12) were obtained from Professor Yuanchao Wang of Nanjing Agricultural University. The isolates were maintained on V8 juice agar slant at 15 °C and transferred to V8 juice agar plate medium at 25 °C for 7 days prior to inoculation. All isolates were tested for virulence against the above differential cultivars using the hypocotyl inoculation technique with mycelium [33]. The isolate virulence formulas and areas of origin are listed in Table 1.

**Table 1:** Virulence formula of 11 isolates of *Phytophthora sojae*

Isolates of *P. sojae*	Virulence formula^a^	Origin
P6497	1b,7	America
HLJ08-17	2,4,5,7	Heilongjiang, China
P7063	1a,1d,3a,6,7	America
AH	2,3a,3b,4,5	Anhui, China,
H15	1b,3b,3c,5,6,7	Heilongjiang, China
HeN08-35	3a,3c,4,5,6,7	Henan, China
PNJ1	1d,2,3b,3c,4,5,7	Jiangsu, China
Pmg	1b,1d,2,3a,3b,4,5,6,7	America
Pm28	1a,1b,1c,1d,1 k,2,3a,3b,3c,5,6,7	America
Pm31	1a,1b,1c,1d,1 k,2,3b,3c,4,5,6,7	America
JS08-12	1a,1b,1c,1d,1 k,2,3a,3b,3c,4,5,6,7	Jiangsu, China

^a^virulence formula shows the resistance gene(s) defeated by an isolate of *P. sojae*

### Plant inoculation and disease assessment

The resistance of the accessions from the soybean mini core collection was evaluated using the hypocotyl inoculation technique with mycelium, which is the preferred method for evaluating soybean resistance to *P. sojae* mediated by *Rps* genes [2]. All screening experiments were conducted at Nanjing Agricultural University in Nanjing, China. Ten seeds of each germplasm accession were planted in a plastic pot filled with vermiculite. Seedling were grown in a greenhouse at 25 °C with a 14-h photoperiod and watered once daily. When its cotyledons fully opened (approximately seven days after planting), the seedlings were prepared for inoculation. An incision (approximately 1 cm in length) was made in the hypocotyl below the cotyledonary node, and a piece of agar (approximately 3 mm square) with mycelium was inoculated into the wound. The inoculated seedlings were placed in a mist room (90 % relative humidity) at 25 °C without light for 10 h and then returned to the greenhouse. The experiment was repeated three times for each isolate to confirm the reactions between *P. sojae* and the seedlings. For each time of inoculation, the differential cultivars were included to verify the success of the inoculation.

Five days after inoculation, accessions were evaluated based on the percentage of survival plants from all replications. Susceptible plants suffered a collapse of the hypocotyl and died, whereas resistant plants developed a hypersensitive reaction (slight necrotic lesions around the wounds). An accession was considered susceptible (S) if less than 30 % of the seedlings survived and was considered resistant (R) if more than 70 % of the seedlings survived. Seedlings survival percentages ranging from 30 % to 70 % were considered intermediate (I) reactions. Intermediate reactions have been broadly classified as resistant reactions according to previous reports [20].

### SNP data

The mini core collection was genotyped with 1,645 SNP markers provided by Professor Lijuan Qiu (listed in Additional file 2); among them, 577 SNPs had already been reported [34]. Minor allele frequency and heterozygosity of markers were assessed using PowerMarker 3.25 software [35].

### Linkage disequilibrium

Pairs of SNPs on the same chromosome were identified as linked SNPs, while those on different chromosomes were identified as unlinked SNPs. The correlation coefficient *r*^2^ was used to estimate the degree of linkage disequilibrium (LD) between each pair of SNPs using TASSEL 5 software [36]. LD decay was evaluated using a nonlinear regression of expected *r*^2^, as described with E (*r*^2^) =1/(1 + 4*N*_e_*c*) [37], where *N*_e_ is the effective population size, and *c* is the recombination rate in Morgan units. We assumed that the soybean genome (Williams 82) and the linkage map had equal sizes (approximately 1.1 GB = 2500 cM) and that 1 cM = 440 Kb between the recombination rates and physical distances. In this way, the physical position of SNP markers was converted to the recombination rate *c*. The *N*_e_ value was estimated using R software (http://www.R-project.org), according to the linear relationship between *c* and *r*^2^. Based on the method developed by Mather [38], the *r*^2^ threshold value fell on the 95th percentile of the unlinked SNPs *r*^*2*^ value.

### Population structure and kinship

STRUCTURE 2.2 software [39] was used to estimate population structure. With an admixture model and an independent allele frequency model, the number of populations (K) was set from 1 to 15 with 5 replications for each K, with the length of the burn-in period set to 10,000 and the number of Monte Carlo Markov Chain (MCMC) replications set to 100,000. K value estimation was determined by the log probability of the data LnP (D) and delta K. Previous studies indicated that there were two or three distinct subgroups in the mini core collection depending on the markers used in the tests [25, 40]. Based on the Q-matrix obtained from the membership probability of each variety, the mini core collection was divided into such subgroups [41].

The kinship matrix [42] was estimated as the genotype similarity between the different pairs of SNPs using TASSEL 5 software [36].

### Association mapping

Association analysis was performed using TASSEL 5 software [36]. Three different models were used to test associations between SNP markers and resistance. The first model was the Naive model, which contained only the SNPs being tested. The second model was a general linear model (GLM) with the Q matrix as a covariate. The third model was a mixed linear model (MLM), where the Q matrix and the relative kinship matrix were included as covariates. The significant association threshold was set to a *P*-value < 10^−3^ (−Log_10_ *P* > 3.00).

### Candidate gene prediction and annotation

Candidate gene positions and functional annotations were retrieved from the Phytozome database (http://www.phytozome.org/) and the Soybase database (http://www.soybase.org/).

## Results

### Resistance reaction to *P. sojae* isolates in the soybean mini core collection

A total of 11 *P. sojae* isolates were used to evaluate the resistance of 224 accessions of the Chinese soybean (*G. max*) mini core collection. The pathotype complexity of the isolates ranged from 2 to 12 (Table 1). The isolates had the most virulence interactions with *Rps*7 (91 %), followed by *Rps*6 (82 %) and *Rps*5 (72 %), and the least virulence interactions with *Rps*1c (27 %) and *Rps*1a (36 %) (Additional file 3).

**Fig. 1:**
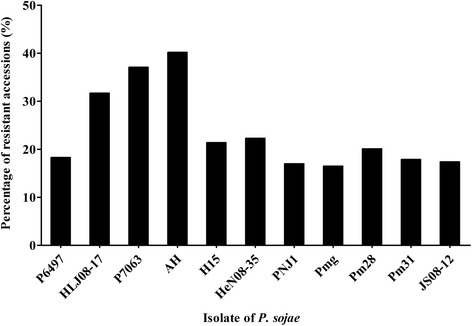
The percentage of resistant accessions to 11 isolates of *P. sojae*

The soybean mini core collection showed varying resistance reactions to the diverse virulence of the isolates. As shown in Fig. 1 and Table 2, more than half of the soybean collection had susceptible reactions to each isolate, which indicates that the soybean in China is extremely susceptible. The largest proportion of soybean germplasm accessions showed up to 40.2 % resistance to the AH isolate. More than half of these resistant accessions came from the Sichuan (13), Hubei (11), Jiangsu (8), Fujian (7), Shandong (6), and Heilongjiang (6) provinces, which are located in South and Huanghuai regions of China. The percentages of accessions with resistance to the P7063 and HLJ08-17 isolates were 37.1 % and 31.7 %, respectively, which ranked second and third. These resistant accessions were mainly from the Hubei (10/13), Jiangsu (9/11), Fujian (8/6), Sichuan (9/3), Shandong (6/5), Hebei (4/6) and Jiangxi (6/6) provinces, which are also located in the South and Huanghuai regions. The percentages of accessions resistant to the other isolates ranged from 22.3 % to 16.5 %. Few accessions (16.5 %) showed resistance to the Pmg isolate, and these came mainly from the Jiangsu (6), Hubei (6), Sichuan (3) and Fujian (3) provinces. In general, the germplasm from the provinces of Jiangsu, Hubei, Sichuan and Fujian in the South and Huanghuai regions of China contributed the top four resistant sources to each isolate (Table 2).

**Table 2:** Geographic distribution of soybean germplasm with resistant to 11 isolates of *P. sojae*

Origin region	Isolate of *P. sojae*										
	P6497	HLJ08-17	P7063	AH	H15	HeN08-35	PNJ1	Pmg	Pm28	Pm31	JS08-12
Jiangsu	6	11	9	8	6	8	6	6	4	4	7
Hubei	8	13	10	11	4	9	6	6	7	5	2
Sichuan	3	3	9	13	4	3	6	3	4	5	2
Fujian	5	6	8	7	5	4	5	3	1	4	3
Jiangxi	1	6	6	2	4	6	1	1	1	3	2
Heilongjiang	1	1	1	6	3	1	1	1	4	7	5
Shandong	2	5	6	6	4	3	2	0	1	0	1
Hebei	1	6	4	2	2	3	1	2	4	1	3
Hunan	3	1	5	5	4	2	1	2	1	1	1
Jilin	2	2	2	4	2	1	2	1	3	2	3
Liaoning	2	3	3	2	1	2	1	2	4	3	2
Shannxi	1	2	4	4	1	2	1	2	1	2	0
Henan	1	1	4	4	1	1	1	1	1	0	1
Guangdong	2	3	3	3	3	1	1	1	3	1	0
Shanxi	1	2	1	5	1	1	1	2	2	1	1
Guizhou	1	0	1	3	0	0	1	2	1	1	0
Guangxi	0	1	3	2	1	0	0	0	1	0	0
Anhui	0	1	2	0	1	1	0	2	1	0	2
Zhejiang	0	2	1	1	0	1	0	0	0	0	2
Hainan	1	1	1	1	1	1	1	0	0	0	0
Xinjiang	0	0	0	0	0	0	0	0	1	0	0
Gansu	0	1	0	0	0	0	0	0	0	0	0
Inner mong	0	0	0	1	0	0	0	0	0	0	0
Beijing	0	0	0	0	0	0	0	0	0	0	1
Yunnan	0	0	0	0	0	0	0	0	0	0	1
Ningxia	0	0	0	0	0	0	0	0	0	0	0
North region	5	6	6	13	6	4	4	4	11	12	10
Northeast region	1	5	2	6	1	1	1	2	5	3	4
Huanghuai region	10	19	24	21	12	14	10	9	8	5	7
South region	25	41	51	50	29	31	23	22	21	20	18

**Fig. 2:**
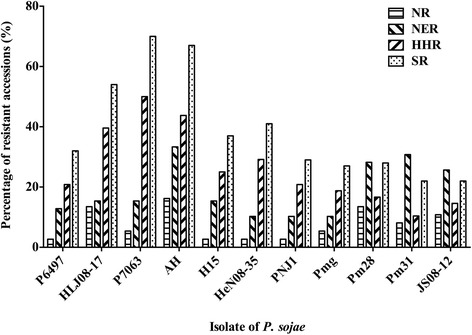
The percentage of resistant accessions in different eco-regions to 11 isolates of *P. sojae*

As shown in Fig. 2, soybean germplasm from the South region (SR) had the highest percentage of resistant accessions. More than 20 % of accessions from the SR showed resistance to each isolate of *P. sojae*, and more than 40 % of accessions showed resistance to the HeN08-35, HLJ08-17, P7063 and AH isolates. This was followed by the soybean germplasm from the Huanghuai region (HHR), for which more than 30 % of accessions were resistant to the AH and Pm31 isolates. The germplasm from the Northeast region (NER) and the North region (NR) had lower percentages of resistant accessions. The most resistant accessions from the NR were resistant to the AH, HLJ08-17 and Pm28 isolates, but all of them had percentages of resistant accessions less than 20 %. Notably, more than 20 % of accessions from the SR and NER were resistant to the Pm28, Pm31 and JS08-12 isolates, which were the most virulent isolates that we used.

### Investigation of the distribution of multi-resistant soybean germplasm in China

**Fig. 3:**
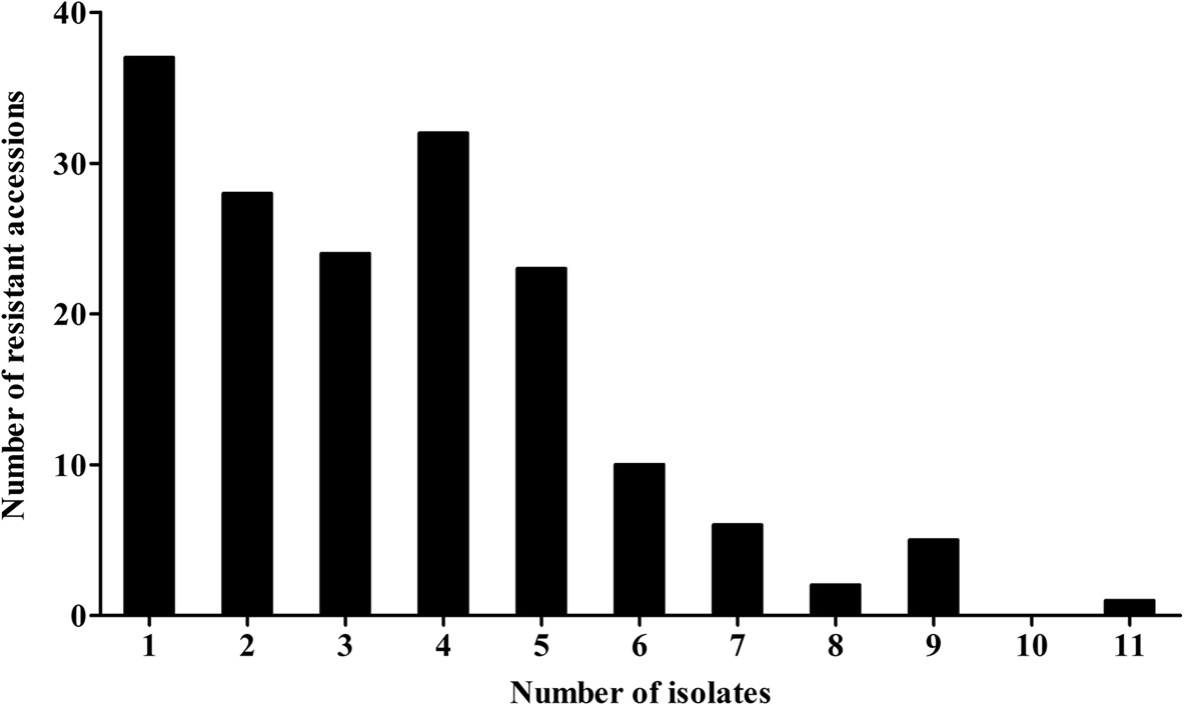
The numbers of resistant accessions to multi-isolates of *P. sojae*

There were 168 (75 %) soybean accessions resistant to 1–11 isolates (Fig. 3 and Table 3). Among them, sixty-five (29.0 %) accessions were resistant to 1–2 isolates, which were concentrated in the Sichuan (7), Shanxi (6), Hebei (6), Shandong (5), Jilin (5) and Liaoning (5) provinces. Fifty-six (25.0 %) accessions were resistant to 3–4 isolates; these accessions were distributed in the Jiangsu (8), Sichuan (7), Heilongjiang (5), Henan (4), Hunan (4) and Jiangxi (4) provinces. Thirty-nine (24.6 %) accessions were resistant to 5–7 isolates. Most of them were concentrated in the Hubei (11) and Fujian (4), Sichuan (3) and Jiangsu (3) provinces. Eight accessions from the Jiangsu (2), Fujian (2), Hubei (1), Hebei (1), Liaoning (1) and Jiangxi (1) provinces were resistant to 8–11 isolates (Table 4); notably, the accession ZDD03776 from the Jiangsu province had the broadest spectrum of resistance, showing resistance to 11 isolates. Overall, most NR soybean accessions showed resistance to less than 4 isolates, and most NER accessions showed resistance to 1–5 isolates. SR and HHR accessions specifically from the Jiangsu, Hubei and Fujian provinces had the most variable and broad-spectrum resistance.

**Table 3:** Geographic distribution of soybean germplasm with resistant to 1–11 *P. sojae* isolates

Origin region	Number of isolates										
	1	2	3	4	5	6	7	8	9	10	11
Ningxia	-	-	-	-	-	-	-	-	-	-	-
Gansu	1	-	-	-	-	-	-	-	-	-	-
Yunnan	1	-	-	-	-	-	-	-	-	-	-
Xinjiang	1	-	-	-	-	-	-	-	-	-	-
Inner mong	1	-	-	-	-	-	-	-	-	-	-
Beijing	1	-	-	-	-	-	-	-	-	-	-
Guangxi	-	1	2	-	-	-		-	-	-	-
Guizhou	2	1	2	-	-	-		-	-	-	-
Zhejiang	-	-	1	1	-	-	-	-	-	-	-
Henan	1	1	3	1	-	-	-	-	-	-	-
Shaanxi	1	2	2	1	1	-	-	-	-	-	-
Anhui	-	-	-	-	2	-	-	-	-	-	-
Heilongjiang	3	1	4	1	2	-	-	-	-	-	-
Hunan	-	-	-	4	2	-		-	-	-	-
Shanxi	3	3	1	-	-	1	-	-	-	-	-
Guangdong	2	2		1	1	1		-	-	-	-
Shandong	2	3	1	2	1	1	-	-	-	-	-
Sichuan	2	5	2	5	1	2	-	-	-	-	-
Hainan	-	-	-	-	-	-	1	-	-	-	-
Jilin	4	1	2	-	1	-	1	-	-	-	-
Jiangxi	1	2	1	3	1	-	-	1	-	-	-
Liaoning	3	2	-	1	1	-	-	-	1	-	-
Hebei	4	2	1	1	1	-	-	-	1	-	-
Hubei	1	1	-	2	7	2	2	-	1	-	-
Fujian	-	-	1	2	2	1	1	1	1	-	-
Jiangsu	3	1	1	7	-	2	1	-	1	-	1
North region	9	5	2	-	-	1	-	-	-	-	-
Northeast region	11	4	6	2	4	-	1	-	1	-	-
Huanghuai region	6	6	6	10	3	2	1	-	2	-	1
South region	11	13	10	20	16	7	4	2	2	-	-

**Table 4:** Accessions with resistant reactions to between 8 to 11 isolates of *Phytophthora sojae*

Accessions	Reaction type^a^	Province	Ecological region
ZDD06501	RRRSRRRRSRS	Jiangxi	South
ZDD21485	RRRRRRSSRSR	Fujian	South
ZDD00921	RRRRRRSRRRS	Liaoning	North
ZDD06363	RRRRRRRRSRS	Fujian	South
ZDD03741	RRRRRRRRSSR	Jiangsu	Huanghuai
ZDD11581	RRRRRRRRRSS	Hubei	South
ZDD18835	RRRRRRRRRSS	Hebei	Huanghuai
ZDD03776	RRRRRRRRRRR	Jiangsu	Huanghuai

^a^The reaction type an accession occurred is a combination of reactions of the accession to P6497, HLJ08-17, P7063, AH, H15, HeN08-35, PNJ1, Pmg, Pm28, Pm31 and JS08-12 isolates

### Postulation of resistance genes in the soybean mini core collection germplasm

There were 109 reaction types to 11 *P. sojae* isolates in the soybean mini core collection (data not shown). By comparing the responses of the mini core collection and 13 differential cultivars, we postulated of gene(s) in the resistant soybean accessions (Table 5). Fifty-six accessions were postulated to have no *Rps* genes with susceptible reactions to all isolates. Seven accessions (ZDD01169, ZDD02149, ZDD04429, ZDD07409, ZDD08018, ZDD17622, and ZDD18524) were postulated to have the *Rps*7 gene because they had the same reaction type (SSSSSSSSSSR) as the differential cultivar Harosoy, which carries *Rps*7. The accessions ZDD14783 and ZDD16675 from the Hunan and Guangdong provinces were postulated to have the *Rps*4 gene because their reaction type RSRRRSSSRSS was consistent with the differential cultivar L85-2352, which carries *Rps*4. The accession ZDD03741 from the Jiangsu province had the reaction type RRRRRRRRSSR and was postulated to have a gene combination of *Rps*1c and *Rps*7. Accession ZDD16874 from the Hainan province had the reaction type RRRRRRRSSSS and was postulated to have gene combinations of *Rps*1b with *Rps*1d/*Rps*2/*Rps*5/*Rps*6. The other 105 reaction types were not consistent with any single *Rps* gene or two-gene combinations. Based on these results, we postulated that more than two *Rps* genes or novel *Rps* genes were present in most resistant accessions.

**Table 5:** Gene postulations and reaction types of soybean accessions

Accession	Reaction type^a^	Gene postulation
ZDD00294, ZDD00310, ZDD00638, ZDD00932, ZDD01402, ZDD01983, ZDD02096. ZDD02114, ZDD02134, ZDD02159, ZDD02315, ZDD02400, ZDD02626, ZDD02940, ZDD03533, ZDD03540, ZDD03733, ZDD06410, ZDD06822, ZDD06823, ZDD07370, ZDD07489, ZDD07623, ZDD08120, ZDD08228, ZDD08238, ZDD08251, ZDD08352, ZDD08472, ZDD08603, ZDD08728, ZDD08928, ZDD08986, ZDD09136, ZDD09279, ZDD10252, ZDD10270, ZDD11092, ZDD11159, ZDD11226, ZDD12407, ZDD12680, ZDD12908, ZDD12910, ZDD13666, ZDD14228, ZDD14920, ZDD16682, ZDD16743, ZDD17325, ZDD17375, ZDD17457, ZDD17574, ZDD18632, ZDD19464, ZDD20671	SSSSSSSSSSS	0
ZDD01169, ZDD02149, ZDD04429, ZDD07409, ZDD08018, ZDD17622, ZDD18524	SSSSSSSSSSR	*Rps*7
ZDD14783, ZDD16675	RSRRRSSSRSS	*Rps*4
ZDD16874	RRRRRRRSSSS	*Rps*1b + *Rps*1d/*Rps*2/ *Rps*5/*Rps*6
ZDD03741	RRRRRRRRSSR	*Rps*1c + *Rps*7

^a^The reaction type an accession occurred is a combination of reactions of the accession to P6497, HLJ08-17, P7063, AH, H15, HeN08-35, PNJ1, Pmg, Pm28, Pm31 and JS08-12 isolates

### Linkage disequilibrium decay in the soybean mini core collection

The soybean mini core collection was genotyped with 1,645 SNP markers. The physical distribution of SNPs was fairly uniform, with an average of 1.72 SNPs/Mb for the entire genome, and varied between a minimum of 0.80 SNPs/Mb on Gm01 and a maximum of 3.57 SNPs/Mb on Gm08 (Additional file 4). After excluding markers with > 15 % missing data, a minor allele frequency < 0.05 and heterozygosity > 0.15, a total of 1,514 high quality SNPs were selected for association mapping.

**Fig. 4:**
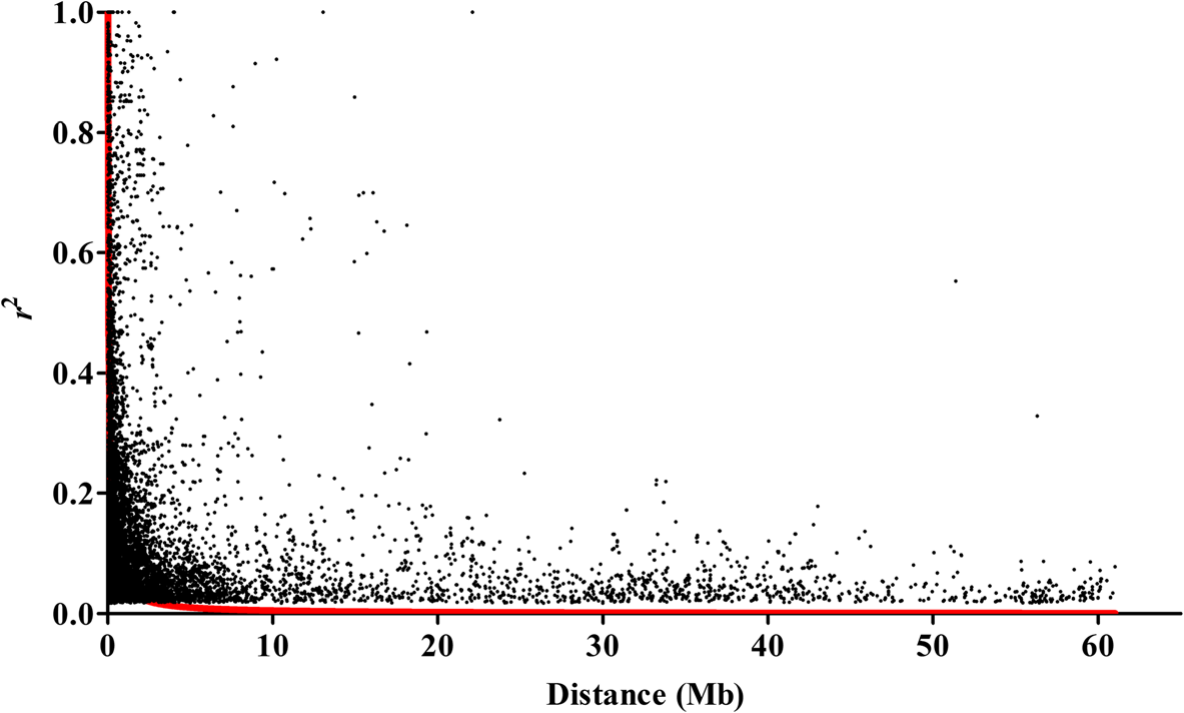
Scatter plots and LD decay against physical distance among co-chromosome SNPs

The mean *r*^2^ values for unlinked and linked SNP pairs were 0.01 and 0.03. With increasing physical distance between loci, *r*^2^ values declined rapidly. The average LD decay for all chromosomes was estimated at 544.01 kb at *r*^2^ < 0.04 and described by the red curve in Fig. 4.

### Population structure and relative kinship of the soybean mini core collection

**Fig. 5:**
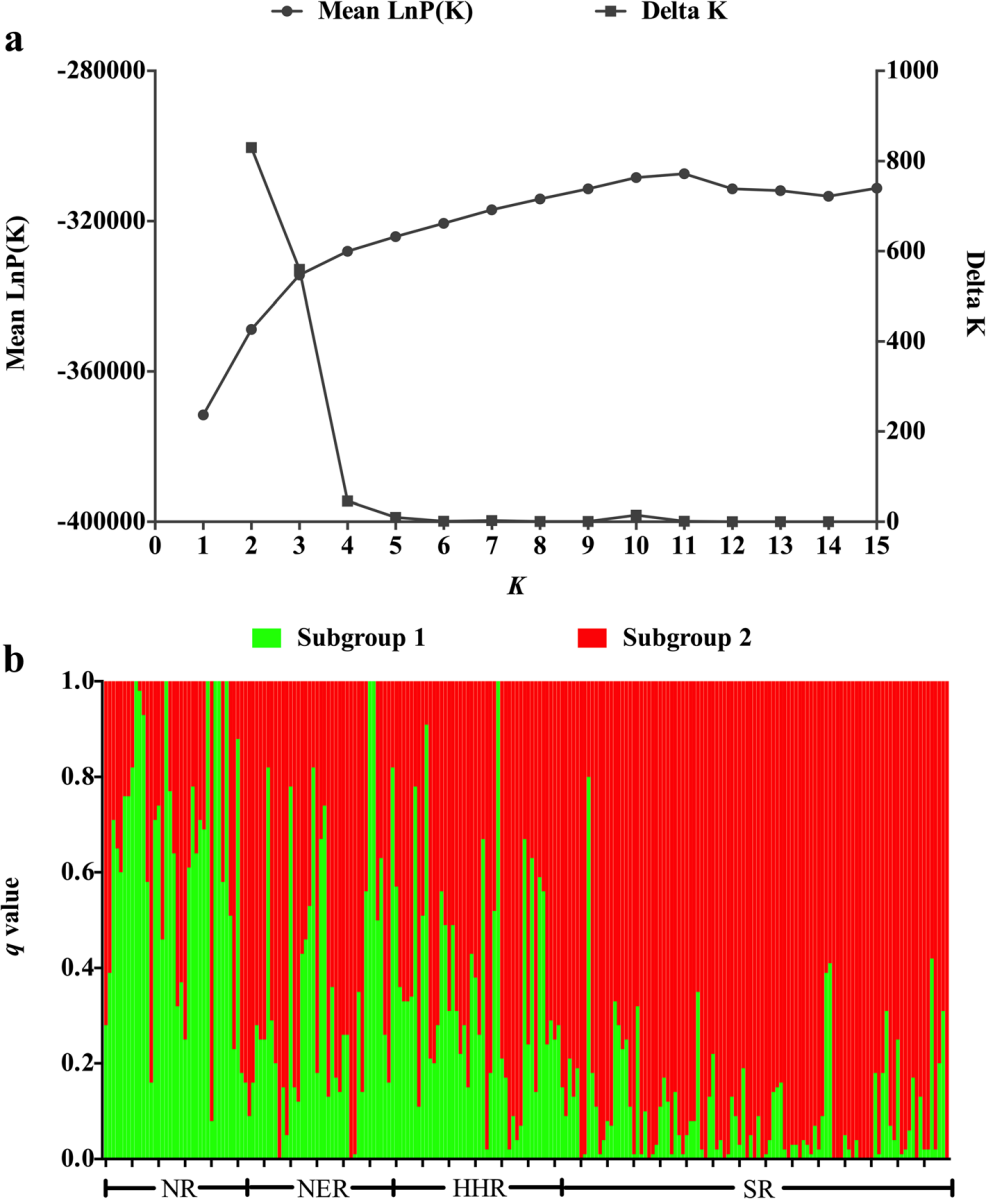
Population structure of the soybean mini core collection. The LnP(D) and Delta K values when k from 1 to 15 shows in Fig (**a**). Fig (**b**) shows the *q* value of soybean accessions belonged to two subgroups. The subgroup 1 shows in green color and subgroup 2 shows in red color. The vertical coordinate of each subgroup indicates the *q* value for each individual, and the digits on the horizontal coordinate represent the origin region of soybean

STRUCTURE 2.2 software [39] analysis indicated that the soybean mini core collection was divided into two main subgroups (Fig. 5a). The germplasm belonging to each subgroup had differences in geographical origin (Fig. 5b). Fifty-one accessions belonged to subgroup 1, among which one accession (ZDD19579) came from the SR, 13 accessions came from the HHR, 27 accessions came from the NR and 10 accessions came from the NER. Subgroup 2 was a large group that included 173 accessions, among which 99 accessions came from the SR, 35 accessions came from the HHR, 10 accessions came from the NR and 29 accessions came from the NER, which accounted for almost all of the germplasm from the SR, 73.0 % from the HHR, 27.0 % from the NR and 74.4 % from the NER. According to the variation and the level of resistance in the four regions, subgroup 2 had the most resistance accessions and a higher resistance level.

**Fig. 6:**
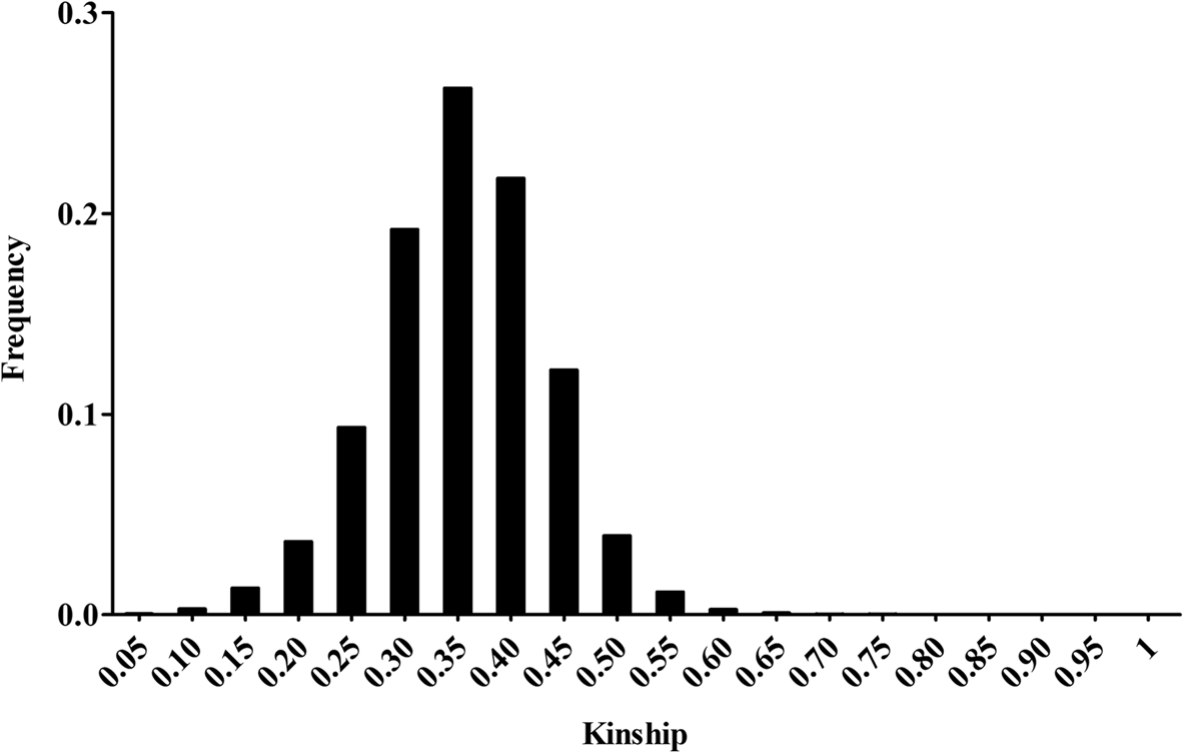
Distribution of pairwise relative kinship estimates between mini core collection

Pairwise kinship was calculated among the soybean accessions. Approximately 67.2 % of the pairwise kinship coefficients were 0.3–0.4, and only 2.0 % were larger than 0.5, indicating weak relatedness among the soybean germplasm (Fig. 6).

### Association mapping and candidate genes for *Phytophthora* resistance

We used a Naive model, a GLM model (Q) and a MLM model (Q + K) for association mapping (Additional file 5). A total of 82 marker-trait associations were detected with the Naive model, while 29 and 12 resistance associations were identified with the GLM and MLM models by applying a *P* < 10^−3^ threshold (Additional file 6). To improve the reliability of the results, we focused on associations supported by at least two models. Finally, fourteen marker-trait associations identified by at least two models were considered to have significant associations with *P. sojae* resistance (Table 6). Nine (64.3 %) of them were within or near the genomic regions detected in previous studies on soybean disease resistance (http://www.soybase.org/), while five associations were newly associated with *Phytophthora* resistance.

**Table 6:** SNP markers identified in association mapping of *P. sojae* resistance

Isolate	SNP markers	Chr	Position	Naive		GLM		MLM		Co-located trait^a^
				-log_10_ *P*	*R* ^*2*^(%)	-log_10_ *P*	*R* ^*2*^(%)	-log_10_ *P*	*R* ^*2*^(%)	
HLJ08-17	Q-15-0369188	15	48863575	5.08	10.35	4.12	8.35	2.85	6.27	New
P7063	Q-03-0266907	3	36634361	5.18	10.43	3.14	5.53	1.82	3.23	SCN [43]
P7063	Q-15-0128012	15	15532390	5.87	13.67	3.60	7.91	2.00	5.05	SCN [44]
H15	Q-16-0268535	16	33793393	2.33	5.02	3.14	6.48	3.03	6.87	BSR [45]
HeN08-35	BARC-014467-01559	16	3962328	3.58	7.48	3.37	6.93	3.17	7.00	New
HeN08-35	BARC-013645-01207	20	46624541	3.34	6.84	3.72	7.46	3.21	6.95	New
PNJ1	BARC-014527-01571	6	644565	3.45	7.28	3.44	7.17	3.44	7.65	SCN [46]
PNJ1	Map-3031	16	15093996	3.04	5.02	3.35	5.54	3.01	5.18	PRR [47, 48]
Pmg	Map-1995	11	7904934	2.84	4.72	3.61	6.16	3.45	6.19	SSR [49]
Pmg	BARC-042413-08254	16	35175092	4.64	9.90	3.97	8.45	3.68	8.33	*Rps*UN2 [50]
Pm28	Q-03-0059953	3	5147782	2.06	3.36	3.19	5.54	3.08	5.69	*Rps*1 [51]
Pm28	BARC-039153-07459	15	831324	5.00	10.67	4.79	10.14	3.68	8.60	PRR [52]
Pm31	Map-0715	4	46749591	1.09	2.35	3.64	7.42	3.09	6.86	New
Pm31	Map-1630	9	3157784	4.37	9.09	3.41	6.98	3.08	6.84	New

^a^The SNP co-located in one of the loci intervals as reported previously

The most significant association with resistance to the HLJ08-35 isolate was on Gm15. The resistance trait for the P7063 isolate had associations in the same region with two SCN-QTLs [43, 44], on Gm03 and Gm15, respectively. One resistance association of the H15 isolate was on Gm16, within a brown stem rot (BSR) resistance QTL [45] interval. For the HeN08-35 isolate, two significant associations were on Gm16 and Gm20, respectively. The resistance to the PNJ1 isolate showed associations with genome regions on Gm06 and Gm16, which were co-located with SCN-QTL [46] and PRR-QTL [47, 48]. For the Pmg isolate, one marker-trait association on Gm11 overlapped with a sclerotinia stem rot (SSR) resistance locus [49], and one on Gm16 in the same *Rps*Un2 interval [50] was identified. Two associations with resistance to the Pm28 isolate were located on Gm03 and Gm15 in the same regions of *Rps*1 [51] and PRR-QTL [52]. Resistance to the Pm31 isolate had two associations, on Gm04 and Gm09.

To shed light on the potential genes involved in resistance to *P. sojae*, 14 significantly associated SNP markers were selected to represent these marker-trait associations (Table 7). They resided in 12 annotated genes, among which 10 SNPs were located in gene exons, and 2 SNPs were located in introns. The candidate genes encode a LEM3 (ligand-effect modulator 3) family/CDC50 family member protein, an ARF-related/ADP-ribosylation factor gene, a predicted lipase class 3 gene, a thioredoxin gene, a DEAD/DEAH box helicase, an oxygenase, a serine/threonine protein kinase, an NPH3 family protein, a zinc finger protein, a lipid transport protein, an ankyrin repeat and a calmodulin-binding motif protein. This is the first time that these genes were reported to be associated with *Phytophthora* resistance.

**Table 7:** Candidate genes related to *P. sojae* resistance

SNP marker	Nucleotide	Location	Gene	Annotation^a^
Q-15-0369188	A/G	Exon	Glyma15g41680	LEM3 (ligand-effect modulator 3) family/CDC50-related
Q-03-0266907	A/G	Exon	Glyma03g28660	ARF-related/ADP-ribosylation factor
Q-15-0128012	A/G	Exon	Glyma15g18620	unknown
Q-16-0268535	A/T	Exon	Glyma16g30140	Predicted lipase class 3 gene
BARC-014467-01559	G/T	Exon	Glyma16g04700	Thioredoxin
BARC-013645-01207	A/G	Exon	Glyma20g39240	DEAD/DEAH box helicase
BARC-014527-01571	C/T	Intron	Glyma06g01080	2OG-Fe (II) oxygenase superfamily
Map-3031	A/G	Intron	Glyma16g14080	Serine/threonine protein kinase
Map-1995	A/G	Exon	Glyma11g11100	Phototropic-responsive NPH3 family protein
BARC-042413-08254	A/G	Exon	Glyma16g31930	Zinc finger domain
Q-03-0059953	A/G	Exon	Glyma03g04960	Lipid transport protein
BARC-039153-07459	A/C	Intron	Glyma15g01370	unknown
Map-0715	A/T	Exon	Glyma04g40800	Serine/threonine protein kinase
Map-1630	C/G	Exon	Glyma09g04310	Ankyrin repeat and calmodulin-binding motif

^a^Annotation description provided by the Soybean Genome Project, DoE Joint Genome Institute (http://www.soybase.org/)

## Discussion

In this study, a systematic and effective analysis of *Phytophthora* resistance to various *P. sojae* isolates in the Chinese soybean mini core collection was performed. The mini core collection was extremely susceptible to the isolates from China and America (Table 1), with an average of 23.6 % of accessions showing resistance to *P. sojae* isolates tested, which implied the urgency of soybean resistance breeding. A total of 168 (75 %) soybean accessions showed resistance to more than one *P. sojae* isolate, suggesting that abundant resistant resources exist. An overview of the resistance spectrum of the mini core collection to 11 *P. sojae* isolates was given. Twenty-four (10.7 %) accessions were resistant to more than five isolates, and eight accessions were notably resistant to 8–11 isolates (Table 4). In conjunction with the previous screening of Chinese resistant germplasm [20, 22, 53], the results provide an excellent database for selection of resistant cultivars and potential breeding materials.

Most accessions resistant to multiple isolates were concentrated in the South and Huanghuai regions, especially in the Jiangsu, Hubei, Sichuan and Fujian provinces, similar to previous results [19, 20, 22, 53, 54]. The South and Huanghuai regions have abundant precipitation (1000–1500 mm/year) and high temperatures (annual accumulated temperature is 4000–8000 °C) [55, 56], which can promote the growth *P. sojae* isolates [9]. The high disease pressure in these regions may be the major factor promoting the variety of resistant resources found therein. The PNJ1 and JS08-12 isolates from the SR and the HeN08-35 isolate from the HHR showed strong virulence to 6–13 *Rps* genes, which coincided with the high level of resistance displayed by soybean in those regions. Conversely, soybean accessions in the NR and NER tend to be more susceptible, resulting in the need to broaden the genetic base and breed resistant cultivars. The results of our study combined with an investigation of *P. sojae* pathotypes, especially the dominant pathotypes from each region of China, may provide a reasonable guide for planting cultivars with different resistance genes, which would be an effective means of PRR disease control.

The various reaction types of soybean accessions indicated that accessions may carry different *Rps* genes. The greater frequency of resistance reactions in the mini core collection than in the differential cultivars implied that the collection may carry more or novel resistance genes. According to the gene postulation, only 67 accessions were predicted to contain *Rps*1b, *Rps*1c, *Rps*4 or *Rps*7. The number of postulated accessions was not as high as in previous studies [20, 22], perhaps due to the complex virulence effects of the isolates we used, which made the probability of a soybean accession having the same reaction type as one differential cultivar only 224/2^13^. The other 157 resistance accessions may have multiple or novel resistance genes. Developing bi-parental populations of these accessions and fine mapping resistance genes may be one way of confirming the results of the gene postulation.

The observed phenotypic variations implied abundant genetic diversity in the mini core collection. When genotyped with 1,645 SNP markers, the mini core collection showed abundant genetic diversity, weak population structure and familial relatedness. We used an association mapping approach to identify genomic loci associated with variations in resistance phenotypes. A total of 14 significant resistance associations were identified, among which five were newly associated with *Phytophthora* resistance, and four were located in known *Rps* gene/QTL regions. Five other associations were located in the same chromosomal regions of resistance QTLs of other diseases, such as SCN and BSR (Table 6), implying that these regions may contain pleiotropic genes or different resistance genes clustered together. Research on these loci may enable understanding of the genetics of resistance to multiple pathogens. The highest *P*-value of significantly associated SNPs was 8.38 × 10^−6^, which is not as high as that of other studies [28, 57]. This may be due to the small number of SNP markers that we used, which may reduce the degree of association. To date, only the *Rps*1k gene has been cloned [58, 59], although the mechanism underlying *Phytophthora* resistance is still unclear. The resistance-associated loci may be helpful in marker-assisted selection and in understanding the mechanism that mediates resistance.

We chose genes where the most significantly associated SNP markers were located as candidate genes for associations. *Glyma15g41680* is a member of the LEM3 (ligand-effect modulator 3) family/CDC50 protein family, and it may be required for phospholipid translocation across the plasma membrane [60]. *Glyma03g28660* is an ADP-ribosylation factor gene. *ADP-ribosylation factor 1* (*RARF1*) induced pathogenesis-related gene expression and pathogen resistance when it was over-expressed in tobacco plants [61], and it acted as a positive regulator of cell death [62]. *Glyma16g30140* is a predicted lipase class 3 gene that may have essential functions in plant defence or priming. Ectopic expression of *AtPAD4* possessing a lipase 3 motif broadened resistance to SCN and root-knot nematode (RKN) diseases in the soybean [63]. *Glyma16g04700* encodes a thioredoxin protein. Two transcript variants of the *Pi-ta* protein, which coupled the original NBS-LRR domain with a C-terminal thioredoxin domain, showed the highest level of expression in comparison to other transcript variants in rice [64]. *Glyma20g39240* is a DEAD/DEAH box helicase, which may take part in splicing the RNA precursor [65] and in transcription initiation [66]. *Glyma06g01080* belongs to the 2OG-Fe (II) oxygenase superfamily and may catalyse the formation of plant hormones, such as ethylene and gibberellins. In *Arabidopsis*, the *DMR6* gene, which has the same domain, is defence-associated and required for susceptibility to downy mildew [67]. *Glyma16g14080* and *Glyma04g40800* encode a serine/threonine protein kinase, which not only has signal transduction activity but also inhibits the release of spores and the germination of the germ tube in *Phytophthora infestans* [68]. *Glyma11g11100* encodes an NPH3 family protein, which activates signal transduction and regulates auxin signalling during plant growth [69, 70]. *Glyma16g31930* contains a zinc finger domain, which has been shown to negatively regulate transcription [71, 72]. Many NBS-LRR domain genes contain zinc finger domains [73], and some have been reported to play a role in resistance to PRR [74]. *Glyma03g04960* encodes a lipid transport protein (LTP); lipid signals are essential for the activation of plant defence responses. Many studies have shown that over-expression of LTPs in plants can enhance their resistance to pathogens [75–77], possibly through inhibition of germination of their spores [78]. *Glyma09g04310* has an ankyrin repeat and a calmodulin-binding motif. The ankyrin repeat participates in the regulation of transcription [79]; for example, the *AKP2* gene in *Arabidopsis* is a negative regulator of *PR-1* (*pathogen-related protein 1*) expression [80]. Calmodulin-binding proteins can detect external stimuli and regulate systemic acquired resistance [81, 82]. Over-expression of *GmCaM4* in the soybean resulted in enhanced resistance to three pathogens through increased expression of pathogenesis-related (PR) genes [83]. In future studies, the functions of these genes in the resistance process will be examined.

Some studies have inferred that some *Rps* genes contain NBS-LRR domains [13, 58, 59]. In our study, no such NBS-LRR-domain gene was found among the candidates, which may due to the complex mechanism of soybean resistance. This situation has been found in other disease studies. For instance, soybean resistance to SCN through the *Rgh1* locus is conferred through copy number variation, but the Glyma18g02680.1 gene in the *Rhg1* locus, which encodes an LRR-kinase, does not contribute to SCN resistance [84, 85].

## Conclusions

The information presented in this study will be of great value to plant breeders in their efforts to develop PRR resistance breeding programs in the soybean. The results revealed the urgency of resistance breeding and provided excellent resources for breeding materials, especially the soybean from the South and Huanghuai regions, which showed high levels of resistance. An association mapping of *Phytophthora* resistance identified fourteen significant marker-trait associations, nine of which were located within known PRR or other disease resistance loci; five of these associations were previously unknown in *Phytophthora* resistance. In the future, candidate genes related to resistance will be functionally identified to understand the resistance mechanism in the soybean.

## Additional files

Additional file 1: The geographic distribution of soybean mini core collection in China. The soybean accessions are divided into four ecological regions: the North region (NR), the Northeast region (NER), the Huanghuai region (HHR) and the South region (SR). (PDF 286 kb) Additional file 2: Information of SNP markers used in association mapping study. (XLS 175 kb) Additional file 3: Percentage of *P. sojae* isolates with a susceptible interaction with *Rps* genes. (PDF 65 kb) Additional file 4: The numbers and distribution of SNPs on each chromosome. (PDF 44 kb) Additional file 5: Genome-wide association study of soybean *P. sojae* resistance in three models. (PDF 2447 kb) Additional file 6: SNP markers associated with *P. sojae* resistance traits in three models. (PDF 76 kb)
