# Supplementary material for: Phenotypic evaluation and genetic dissection of resistance to Phytophthora sojae in the Chinese soybean mini core collection
Source: BMC Genet. 2016 Jun 18;17:85. doi: 10.1186/s12863-016-0383-4 (PMC4912746; doi:10.1186/s12863-016-0383-4)

Additional files 1 The geographic distribution of soybean mini core collection in China. The soybean accessions are divided into four ecological regions: the North region (NR), the Northeast region (NER), the Huanghuai region (HHR) and the South region (SR).

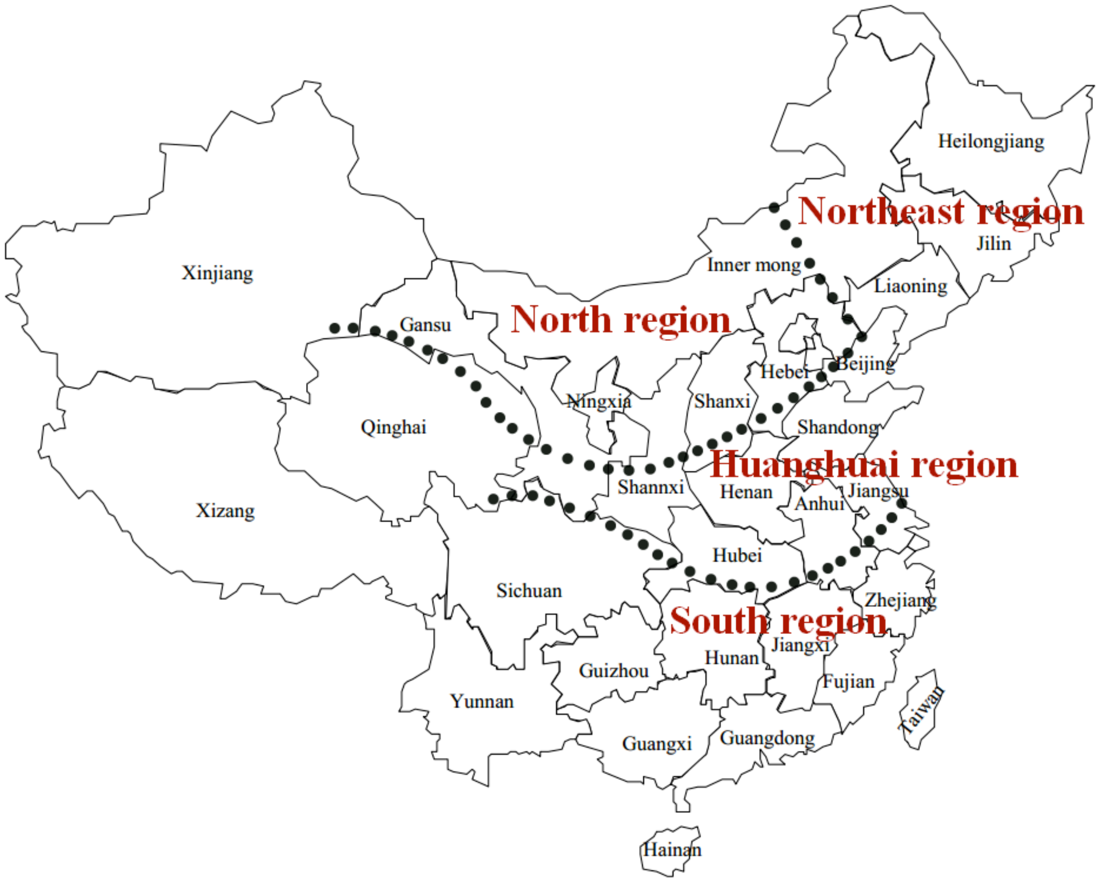

Additional files 1 The geographic distribution of soybean mini core collection in China. The soybean accessions are divided into four ecological regions: North region (NR), Northeast region (NER), Huanghuai region (HHR) and South region (SR).

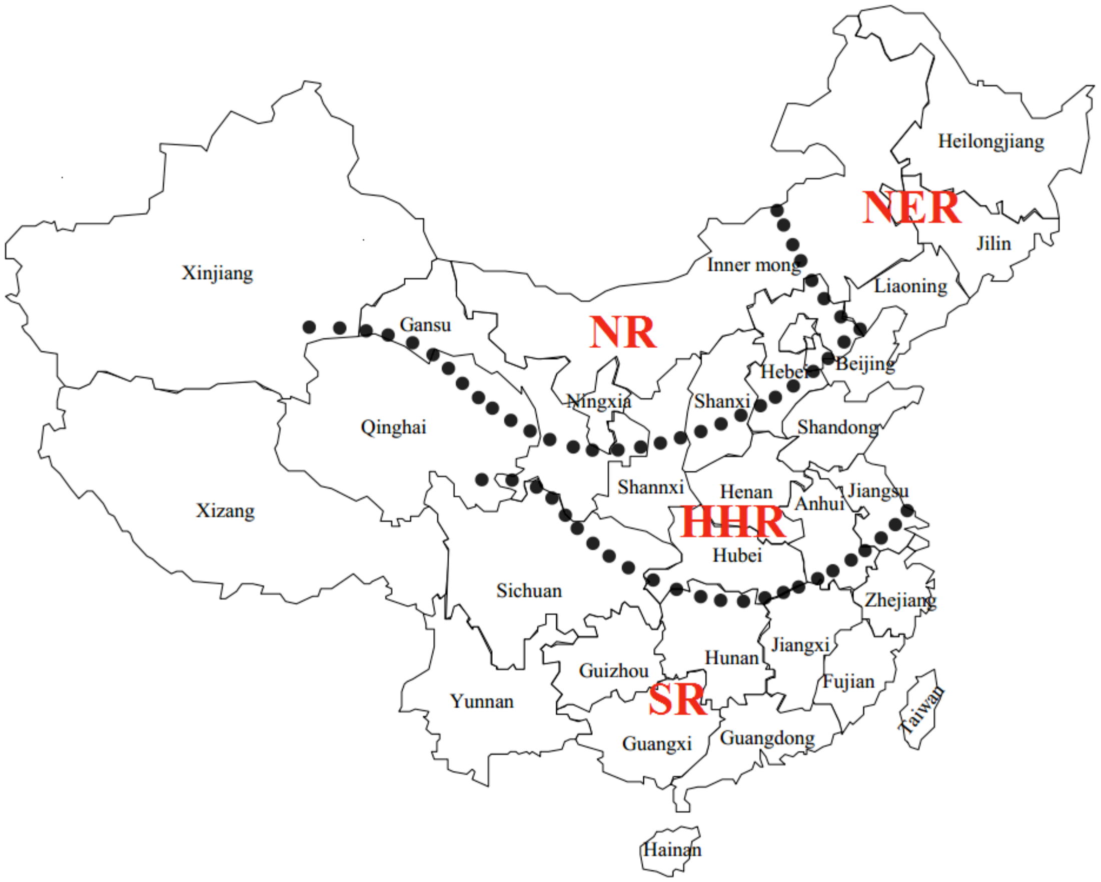

Supplement: Additional file 1: — The geographic distribution of soybean mini core collection in China. The soybean accessions are divided into four ecological regions: the North region (NR), the Northeast region (NER), the Huanghuai region (HHR) and the South region (SR). (PDF 286 kb) [file 12863_2016_383_MOESM1_ESM.pdf]
