# Supplementary material for: Phenotypic evaluation and genetic dissection of resistance to Phytophthora sojae in the Chinese soybean mini core collection
Source: BMC Genet. 2016 Jun 18;17:85. doi: 10.1186/s12863-016-0383-4 (PMC4912746; doi:10.1186/s12863-016-0383-4)

Additional files 3 Percentage of *P. sojae* isolates with a susceptible interaction with *Rps* genes

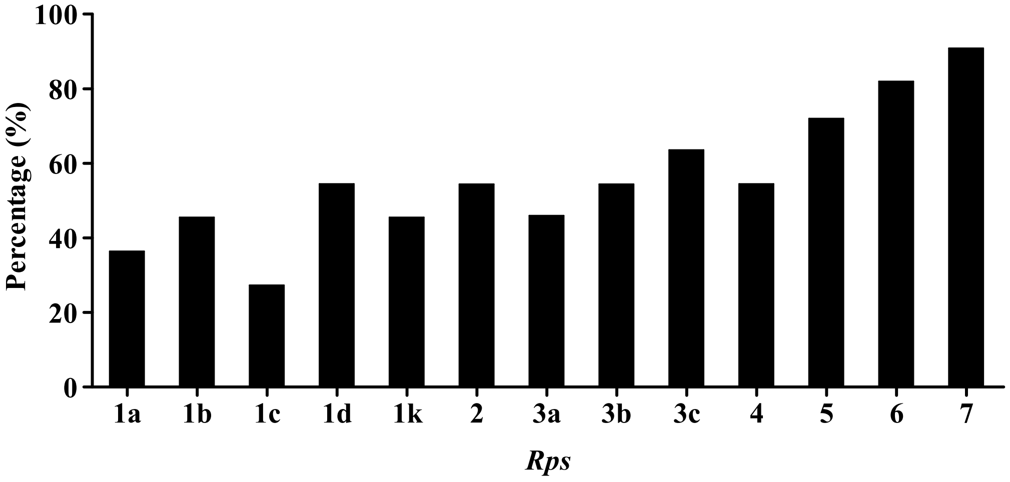

Supplement: Additional file 3: — Percentage of P. sojae isolates with a susceptible interaction with Rps genes. (PDF 65 kb) [file 12863_2016_383_MOESM3_ESM.pdf]
