# Supplementary material for: Phenotypic evaluation and genetic dissection of resistance to Phytophthora sojae in the Chinese soybean mini core collection
Source: BMC Genet. 2016 Jun 18;17:85. doi: 10.1186/s12863-016-0383-4 (PMC4912746; doi:10.1186/s12863-016-0383-4)
Supplement: Additional file 4: — The numbers and distribution of SNPs on each chromosome. (PDF 44 kb) [file 12863_2016_383_MOESM4_ESM.pdf]

Additional files 4 The number and distribution of SNP markers on each chromosome

Chromosome	Num of SNPs	Length(bp)	Kbs/SNP	SNPs/Mb
Gm01	45	55915595	1242.57	0.80
Gm02	65	51656713	794.72	1.26
Gm03	49	47781076	975.12	1.03
Gm04	49	49243852	1004.98	1.00
Gm05	97	41936504	432.34	2.31
Gm06	61	50722821	831.52	1.20
Gm07	123	44683157	363.28	2.75
Gm08	168	46995532	279.74	3.57
Gm09	72	46843750	650.61	1.54
Gm10	42	50969635	1213.56	0.82
Gm11	50	39172790	783.46	1.28
Gm12	34	40113140	1179.80	0.85
Gm13	66	44408971	672.86	1.49
Gm14	123	49711204	404.16	2.47
Gm15	143	50939160	356.22	2.81
Gm16	62	37397385	603.18	1.66
Gm17	68	41906774	616.28	1.62
Gm18	202	62308140	308.46	3.24
Gm19	49	50589441	1032.44	0.97
Gm20	78	46773167	599.66	1.67
Mean	82.3		717.25	1.72
