# Supplementary material for: Phenotypic evaluation and genetic dissection of resistance to Phytophthora sojae in the Chinese soybean mini core collection
Source: BMC Genet. 2016 Jun 18;17:85. doi: 10.1186/s12863-016-0383-4 (PMC4912746; doi:10.1186/s12863-016-0383-4)
Supplement: Additional file 5: — Genome-wide association study of soybean P. sojae resistance in three models. (PDF 2447 kb) [file 12863_2016_383_MOESM5_ESM.pdf]

Additional files 5 Genome-wide association study of soybean *P. sojae* resistance in three models

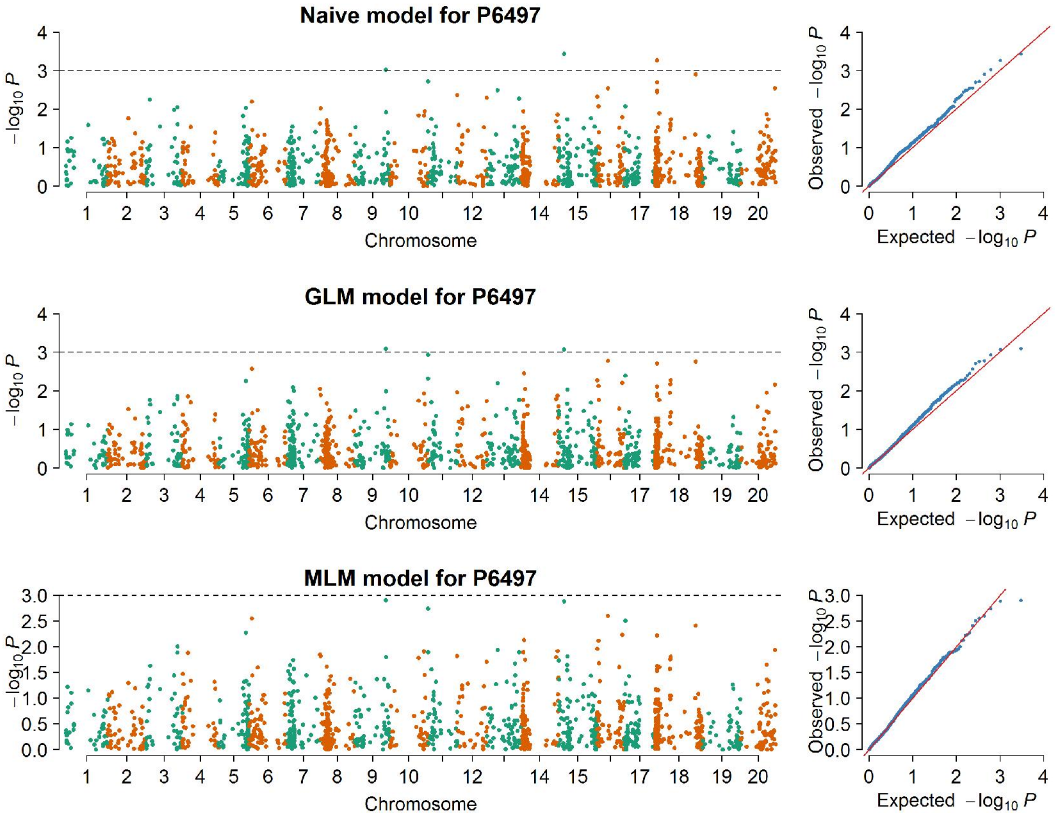

(A) Manhattan plots and Quantile-quantile (Q-Q) plots of resistance to P6497 isolate in three models

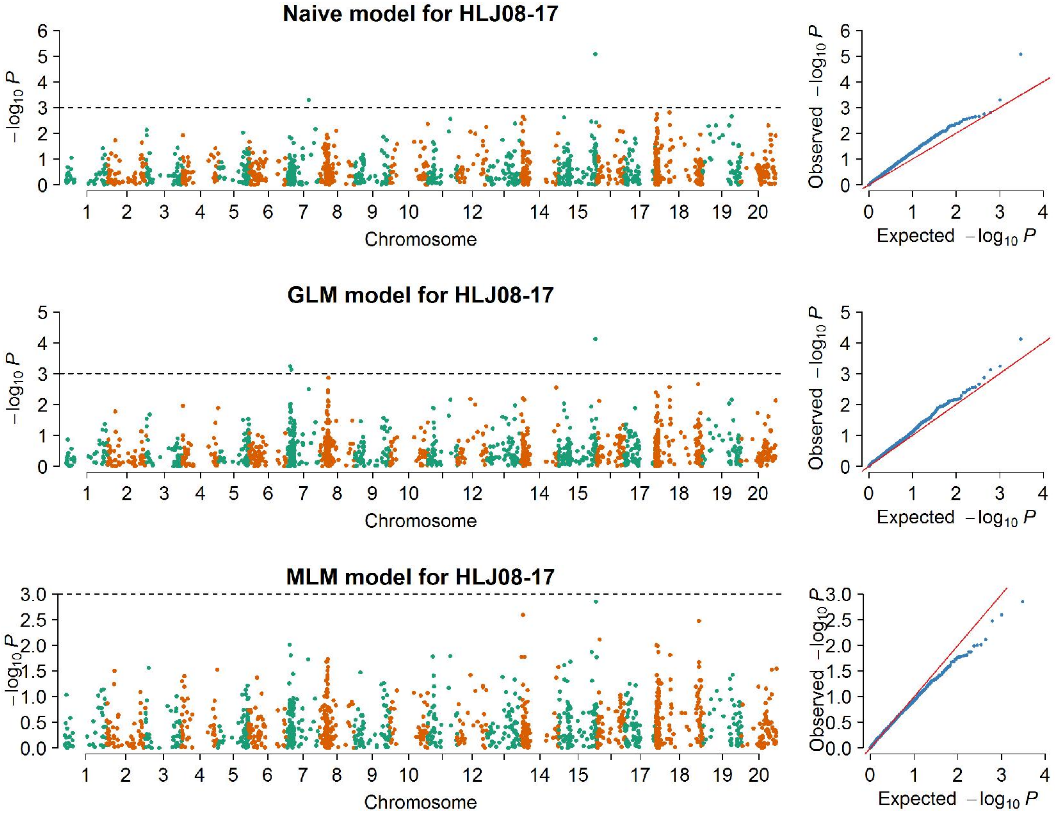

(B) Manhattan plots and Quantile-quantile (Q-Q) plots of resistance to HLJ08-17 isolate in three models

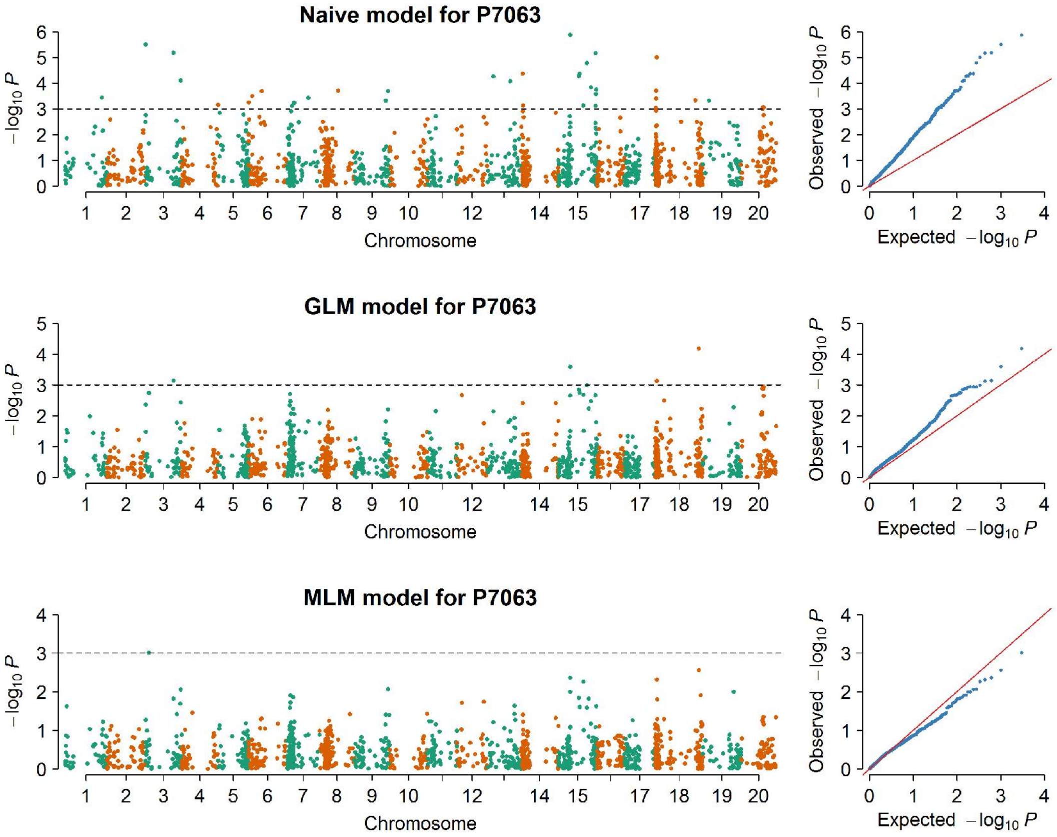

(C) Manhattan plots and Quantile-quantile (Q-Q) plots of resistance to P7063 isolate in three models

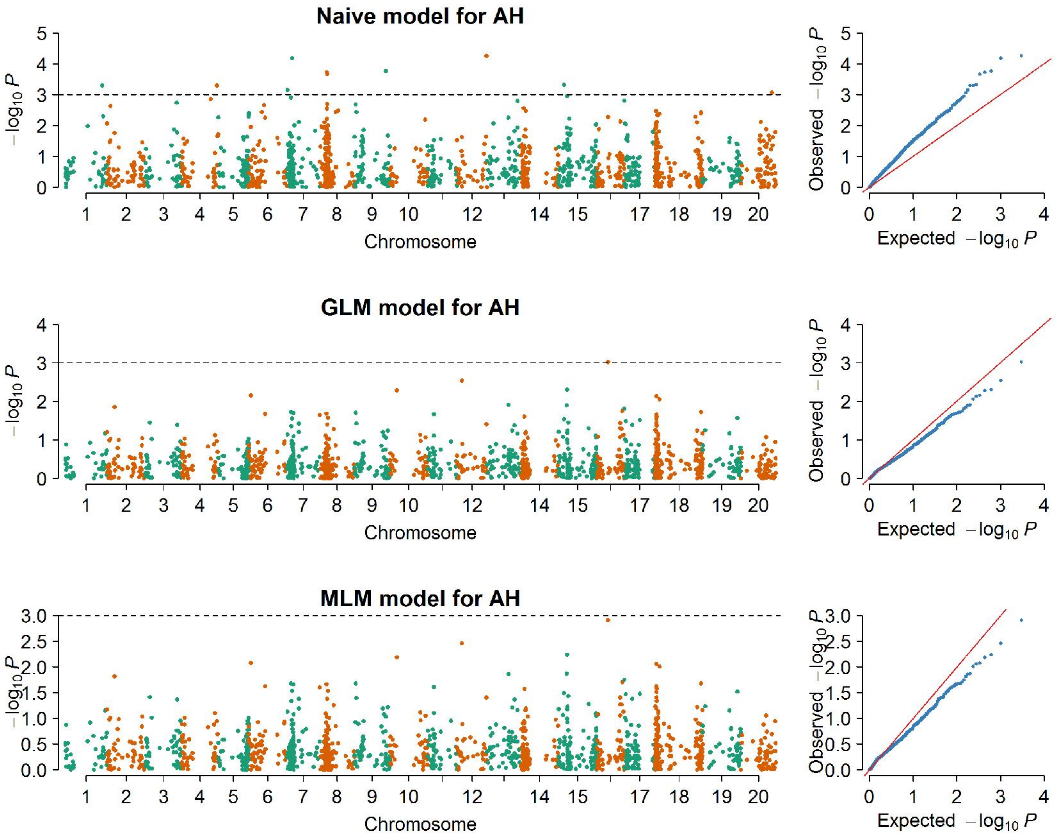

(D) Manhattan plots and Quantile-quantile (Q-Q) plots of resistance to AH isolate in three models

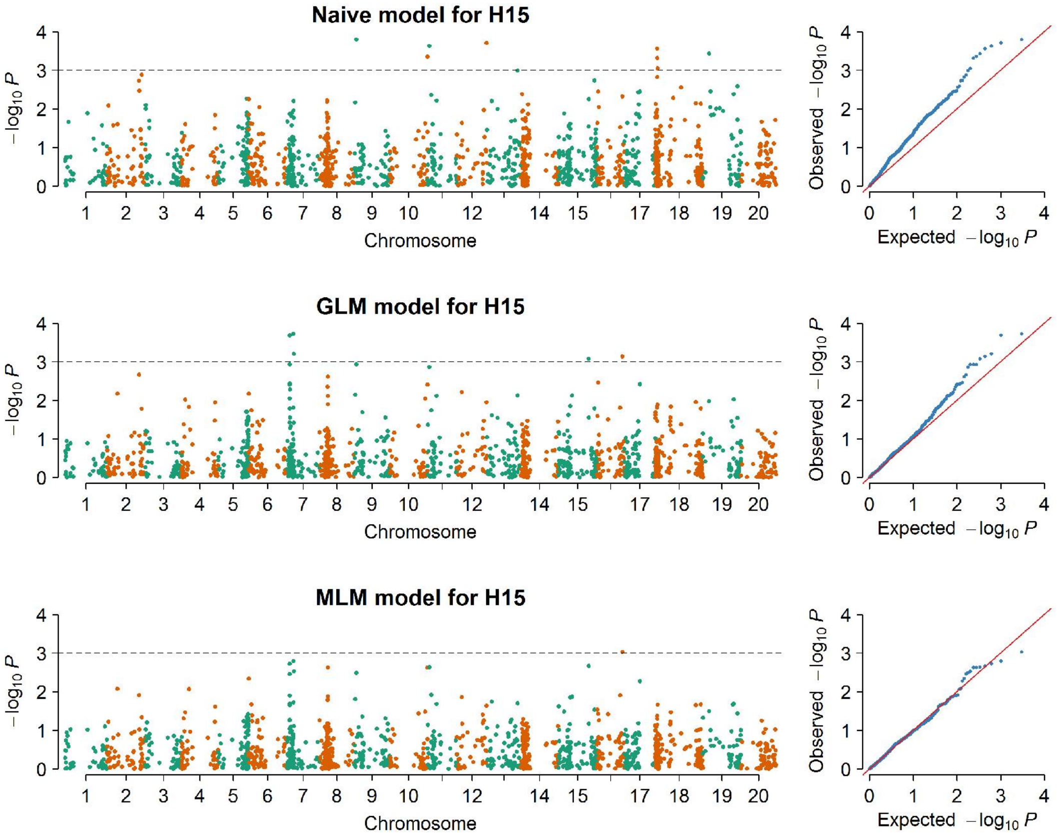

(E) Manhattan plots and Quantile-quantile (Q-Q) plots of resistance to H15 isolate in three models

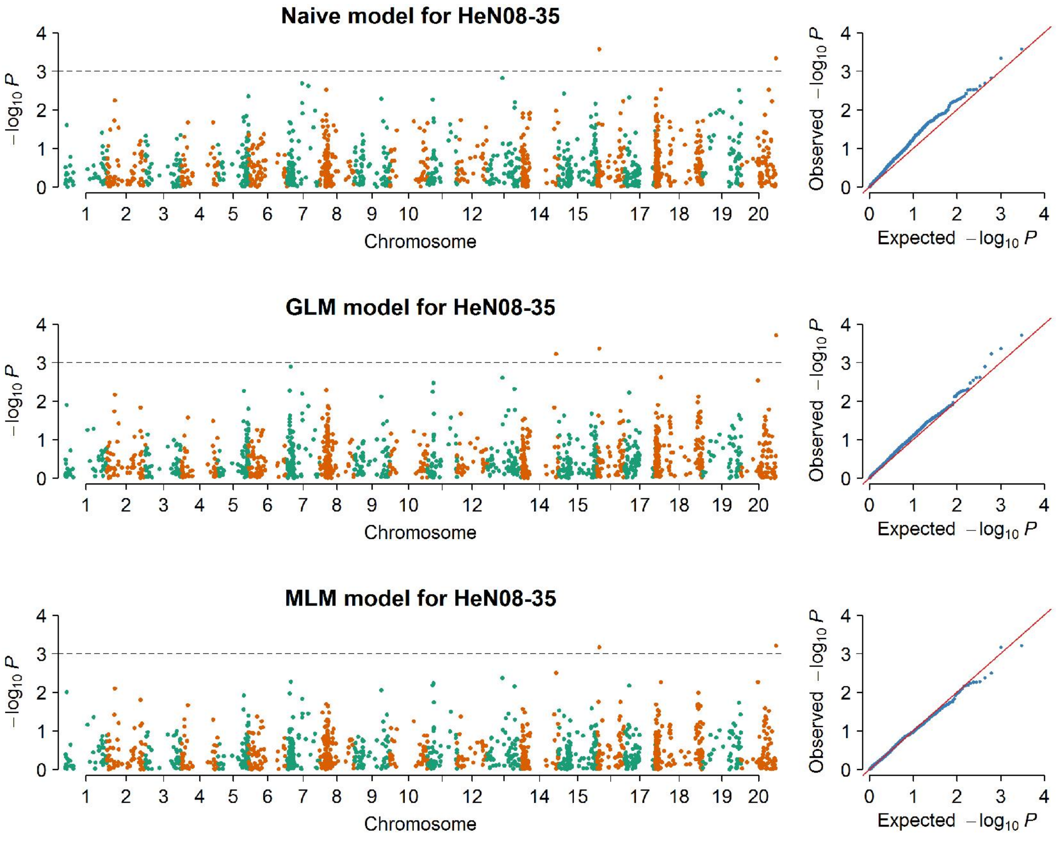

(F) Manhattan plots and Quantile-quantile (Q-Q) plots of resistance to HeN08-35 isolate in three models

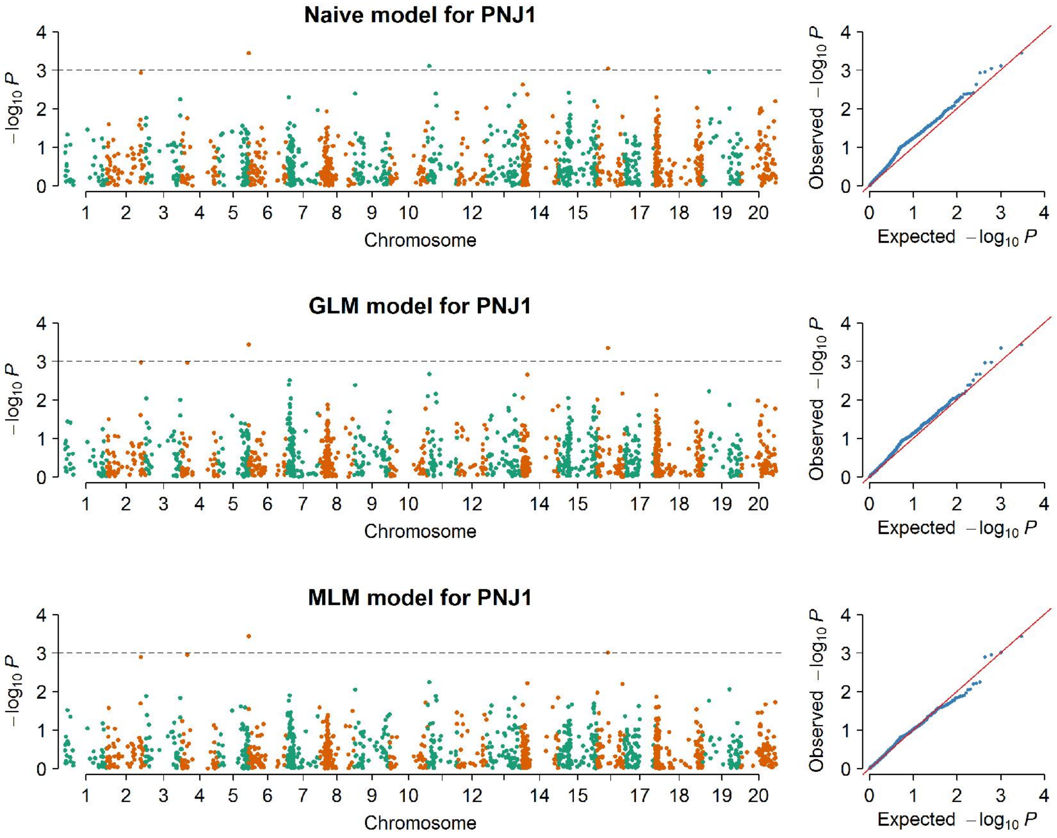

(G) Manhattan plots and Quantile-quantile (Q-Q) plots of resistance to PNJ1 isolate in three models

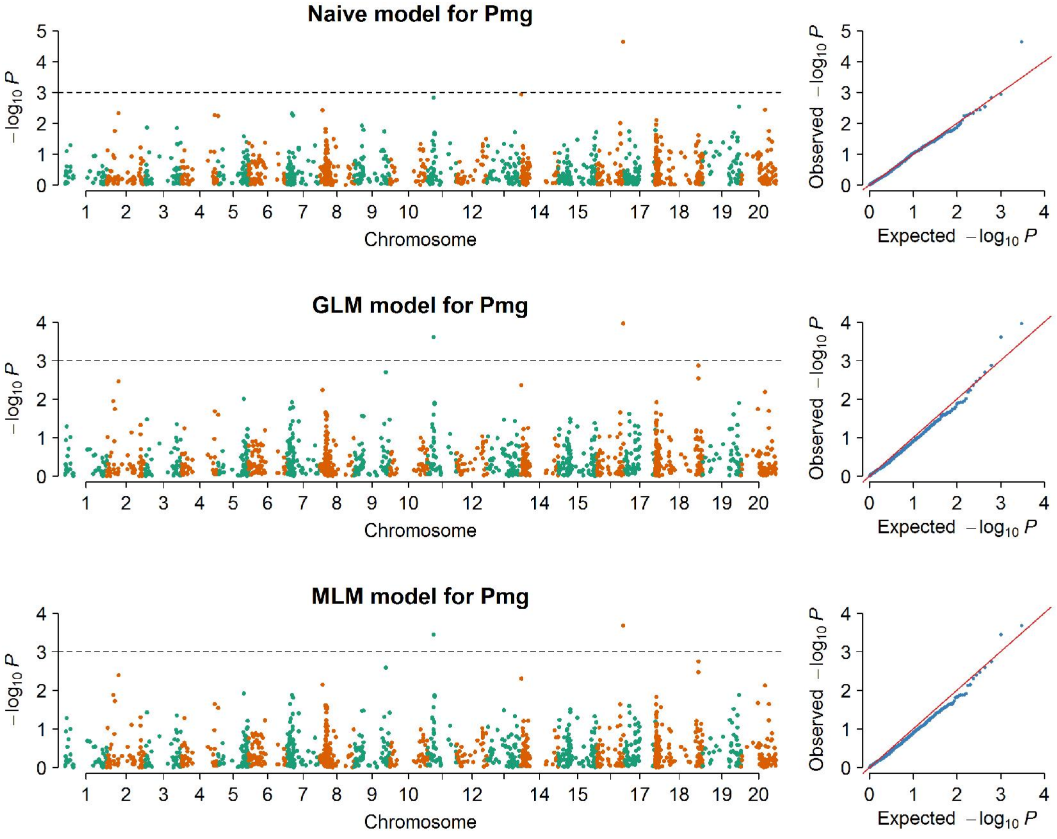

(H) Manhattan plots and Quantile-quantile (Q-Q) plots of resistance to Pmg isolate in three models

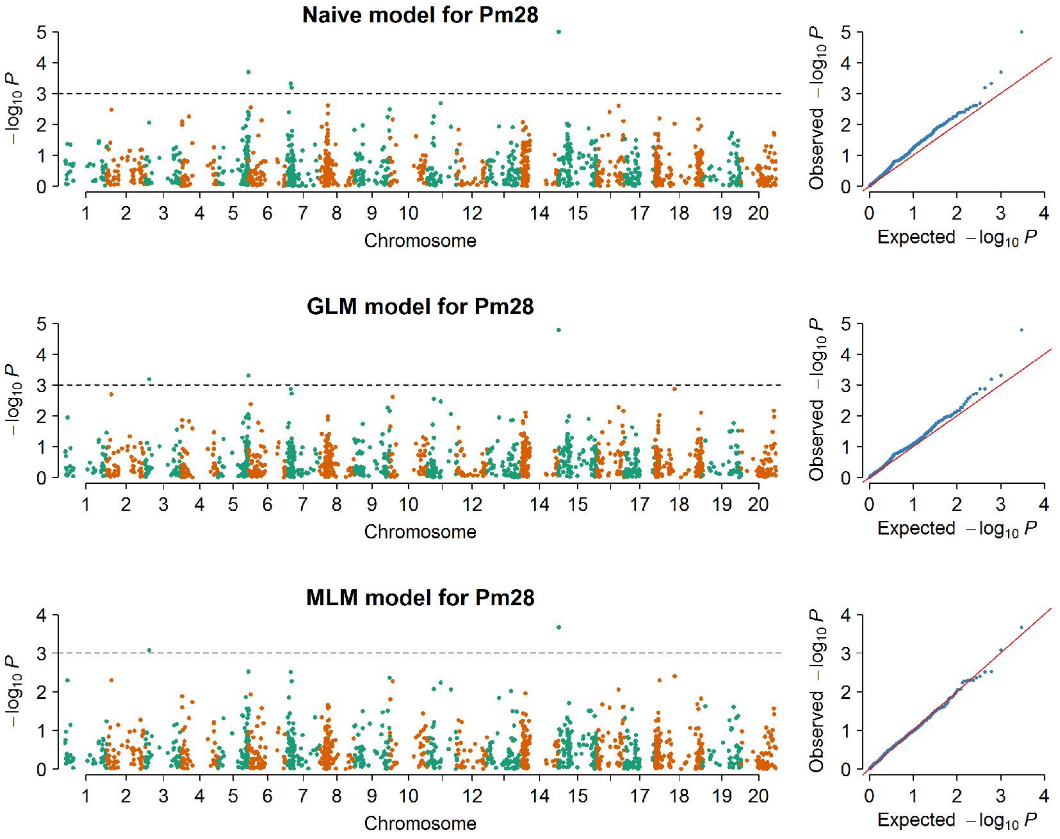

(I) Manhattan plots and Quantile-quantile (Q-Q) plots of resistance to Pm28 isolate in three models

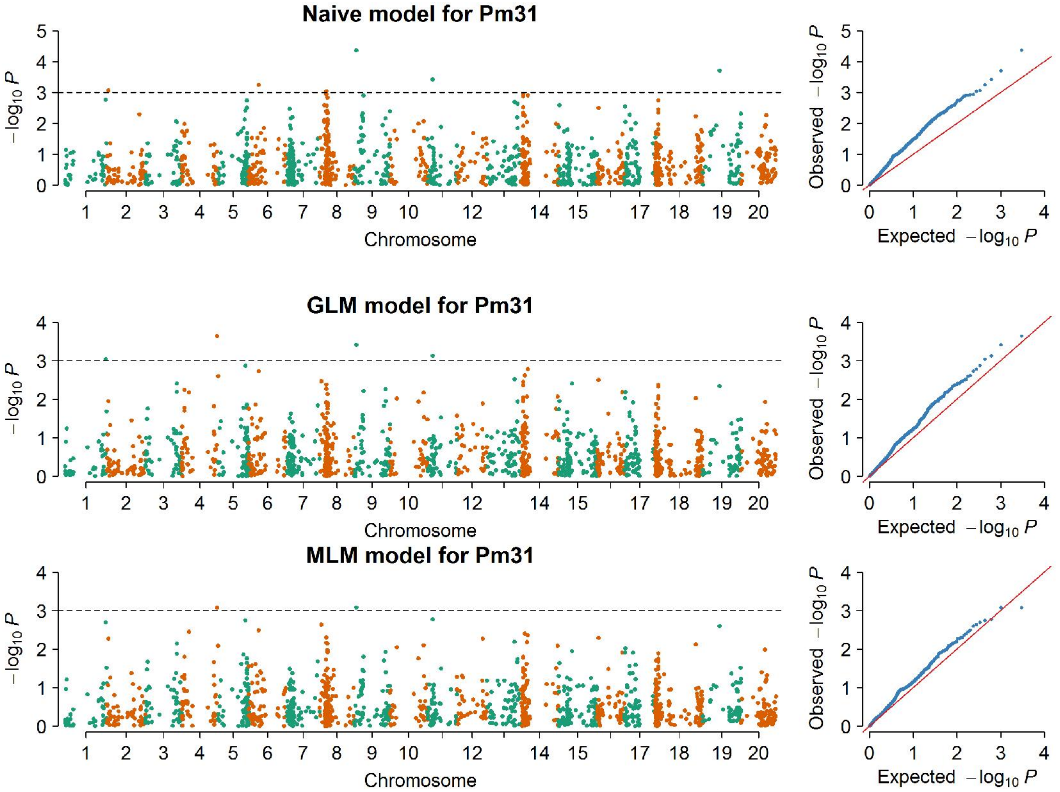

(J) Manhattan plots and Quantile-quantile (Q-Q) plots of resistance to Pm31 isolate in three models

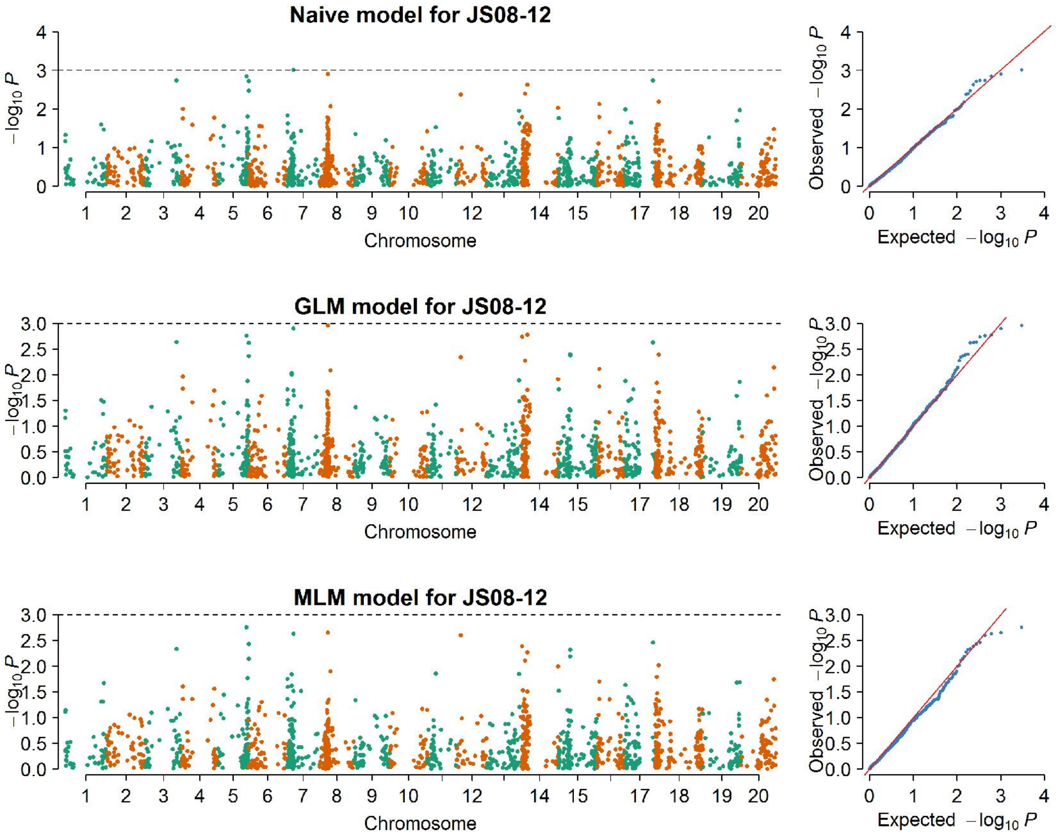

(K) Manhattan plots and Quantile-quantile (Q-Q) plots of resistance to JS08-12 isolate in three models
