# Supplementary material for: Phenotypic evaluation and genetic dissection of resistance to Phytophthora sojae in the Chinese soybean mini core collection
Source: BMC Genet. 2016 Jun 18;17:85. doi: 10.1186/s12863-016-0383-4 (PMC4912746; doi:10.1186/s12863-016-0383-4)
Supplement: Additional file 6: — SNP markers associated with P. sojae resistance traits in three models. (PDF 76 kb) [file 12863_2016_383_MOESM6_ESM.pdf]

Additional file 6 SNP markers associated with *P. sojae* resistance traits in three models

A: Naive model

Isolate	SNP	Chromosome	Position	$-\log_{10}P$	R^2(%)
P6497	BARC-030457-06870	9	41184482	3.02	6.69
P6497	BARC-025753-05037	15	7513319	3.44	7.50
P6497	Q-18-0023952	18	2630774	3.27	5.66
HLJ08-17	Map-1344	7	27907282	3.30	5.51
HLJ08-17	Q-15-0369188	15	48863575	5.08	10.35
P7063	BARC-044221-08647	1	48362058	3.45	8.40
P7063	Map-0359	3	283046	5.50	13.07
P7063	Q-03-0266907	3	36634361	5.18	10.43
P7063	Q-03-0335834	3	45730918	4.11	8.20
P7063	Map-0721	4	48070478	3.16	7.61
P7063	Q-06-0006377	6	910543	3.25	7.82
P7063	BARC-024049-04718	6	5375696	3.49	8.60
P7063	Q-06-0128380	6	17559144	3.70	8.84
P7063	Q-07-0097016	7	9502829	3.25	6.25
P7063	BARC-039195-07466	7	6528808	3.12	5.96
P7063	Q-07-0097039	7	9504770	3.22	6.18
P7063	Map-1344	7	27907282	3.43	6.67
P7063	Q-08-0188076	8	25110324	3.71	7.24
P7063	BARC-025669-04989	9	41620640	3.32	8.11
P7063	BARC-018515-02927	9	44398393	3.69	9.02
P7063	BARC-018551-02971	13	8529479	4.27	10.29
P7063	BARC-014579-01586	13	30619983	4.08	9.85
P7063	Q-14-0026827	14	3162634	4.37	8.72
P7063	Q-14-0034437	14	3636340	3.13	7.53
P7063	Q-15-0128012	15	15532390	5.87	13.67
P7063	Map-2857	15	26869986	4.28	10.16
P7063	Map-2858	15	28157599	4.38	10.43
P7063	Map-2860	15	32720433	3.13	6.01
P7063	Map-2878	15	37092272	4.79	11.66
P7063	Q-15-0301740	15	42869772	3.84	7.58
P7063	Q-15-0368870	15	48837331	3.12	7.52
P7063	Q-15-0369188	15	48863575	5.17	12.14
P7063	Q-15-0373491	15	49278068	3.58	8.57

P7063	Q-15-0373802	15	49297953	3.58	8.57
P7063	Map-2946	15	49563606	3.76	7.63
P7063	Q-18-0004745	18	649027	3.05	5.80
P7063	Q-18-0008588	18	1074902	3.71	7.24
P7063	Q-18-0010221	18	1296260	3.42	6.94
P7063	Q-18-0018797	18	2093358	5.01	11.79
P7063	BARC-029457-06193	18	52346972	3.34	8.19
P7063	Map-3846	19	9119028	3.33	8.16
P7063	Q-20-0141902	20	28765452	3.00	5.74
P7063	Q-20-0143665	20	29057745	3.06	5.83
P7063	Q-20-0149811	20	29985714	3.03	5.78
P7063	Q-20-0150254	20	30044963	3.06	5.83
P7063	Q-20-0150256	20	30045086	3.06	5.83
P7063	Q-20-0151067	20	30182760	3.06	5.83
AH	BARC-044221-08647	1	48362058	3.30	7.29
AH	Map-0703	4	46288957	3.31	7.23
AH	Q-07-0003086	7	491568	3.16	5.44
AH	BARC-039195-07466	7	6528808	4.18	7.45
AH	Q-08-0072666	8	9966020	3.73	6.56
AH	Q-08-0077088	8	10546300	3.66	6.44
AH	BARC-025669-04989	9	41620640	3.77	8.28
AH	Map-2304	12	39133413	4.26	7.75
AH	BARC-025753-05037	15	7513319	3.32	7.32
AH	Map-4219	20	41116710	3.07	6.82
H15	Map-1630	9	3157784	3.80	8.08
H15	BARC-041935-08142	10	49905877	3.36	7.41
H15	Q-11-0016644	11	2541677	3.63	7.72
H15	Map-2304	12	39133413	3.71	6.53
H15	Q-18-0026387	18	2900614	3.06	6.54
H15	Q-18-0023829	18	2613537	3.32	7.10
H15	Q-18-0023952	18	2630774	3.57	6.22
H15	Map-3846	19	9119028	3.43	7.46
HeN08-35	BARC-014467-01559	16	3962328	3.58	7.48
HeN08-35	BARC-013645-01207	20	46624541	3.34	6.84
PNJ1	BARC-014527-01571	6	644565	3.45	7.28
PNJ1	Q-11-0016644	11	2541677	3.11	6.47

PNJ1	Map-3031	16	15093996	3.04	5.02
Pmg	BARC-042413-08254	16	35175092	4.64	9.90
Pm28	Q-05-0215089	5	41180396	3.69	7.89
Pm28	Q-07-0044420	7	5319998	3.32	7.08
Pm28	Q-07-0057212	7	6232512	3.19	6.81
Pm28	BARC-039153-07459	15	831324	5.00	10.67
Pm31	Q-02-0014996	2	2393918	3.07	6.48
Pm31	BARC-028177-05786	6	13550856	3.25	6.97
Pm31	Q-08-0069023	8	9534794	3.04	6.45
Pm31	Map-1630	9	3157784	4.37	9.09
Pm31	BARC-030313-06852	11	6860672	3.42	7.37
Pm31	Map-3887	19	22852602	3.70	6.37
JS08-12	Q-07-0087442	7	8685950	3.02	4.98

B: GLM model

Isolate	SNP	Chromosome	Position	$-\log_{10}P$	R^2(%)
P6497	BARC-030457-06870	9	41184482	3.09	6.78
P6497	BARC-025753-05037	15	7513319	3.07	6.67
HLJ08-17	BARC-013845-01256	7	3598762	3.24	6.74
HLJ08-17	Q-07-0040795	7	5035046	3.13	5.08
HLJ08-17	Q-15-0369188	15	48863575	4.12	8.35
P7063	Q-03-0266907	3	36634361	3.14	5.53
P7063	Q-15-0128012	15	15532390	3.6	7.91
P7063	Q-18-0018797	18	2093358	3.13	6.92
P7063	BARC-021407-04097	18	56723653	4.19	9.39
AH	Map-3031	16	15093996	3.03	4.82
H15	Q-07-0027821	7	3606751	3.69	7.57
H15	Q-07-0088101	7	8730657	3.73	6.3
H15	Q-07-0092431	7	9128285	3.21	5.28
H15	Q-16-0268535	16	33793393	3.14	6.48
H15	Map-2890	15	39992796	3.08	6.42
HeN08-35	Map-2655	14	46842767	3.23	5.22
HeN08-35	BARC-014467-01559	16	3962328	3.37	6.93
HeN08-35	BARC-013645-01207	20	46624541	3.72	7.46
PNJ1	BARC-014527-01571	6	644565	3.44	7.17
PNJ1	Map-3031	16	15093996	3.35	5.54

Pmg	Map-1995	11	7904934	3.61	6.16
Pmg	BARC-042413-08254	16	35175092	3.97	8.45
Pm28	Q-03-0059953	3	5147782	3.19	5.54
Pm28	Q-05-0215089	5	41180396	3.31	7.03
Pm28	BARC-039153-07459	15	831324	4.79	10.14
Pm31	Map-0143	1	53579970	3.05	6.26
Pm31	Map-0715	4	46749591	3.64	7.42
Pm31	Map-1630	9	3157784	3.41	6.98
Pm31	BARC-030313-06852	11	6860672	3.13	6.57

C: MLM model

Isolate	SNP	Chromosome	Position	$-\log_{10}P$	R^2(%)
P7063	Q-03-0049678	3	4487138	3.02	7.72
H15	Q-16-0268535	16	33793393	3.03	6.87
HeN08-35	BARC-013645-01207	20	46624541	3.21	6.95
HeN08-35	BARC-014467-01559	16	3962328	3.17	7.00
PNJ1	BARC-014527-01571	6	644565	3.44	7.65
PNJ1	Map-3031	16	15093996	3.01	5.18
Pmg	Map-1995	11	7904934	3.45	6.19
Pmg	BARC-042413-08254	16	35175092	3.68	8.33
Pm28	Q-03-0059953	3	5147782	3.08	5.68
Pm28	BARC-039153-07459	15	831324	3.68	8.60
Pm31	Map-0715	4	46749591	3.09	6.86
Pm31	Map-1630	9	3157784	3.08	6.84
